# Fasciculation distribution in a healthy population assessed with diffusion tensor imaging

**DOI:** 10.14814/phy2.70247

**Published:** 2025-03-22

**Authors:** Linda Heskamp, Lara Schlaffke, Johannes Forsting, Boudewijn T. H. M. Sleutjes, H. Stephan Goedee, Martijn Froeling

**Affiliations:** ^1^ Center for Image Sciences, Precision Imaging Group, Division Imaging & Oncology University Medical Centre Utrecht Utrecht The Netherlands; ^2^ Department of Neurology BG‐University Hospital Bergmannsheil gGmbH Bochum Germany; ^3^ Department of Neurology and Neurosurgery, Brain center University Medical Centre Utrecht Utrecht The Netherlands

**Keywords:** diffusion tensor imaging, fasciculation, magnetic resonance imaging, motor unit, skeletal muscle

## Abstract

Fasciculations, a hallmark of motor neuron diseases, also occur in healthy individuals, highlighting the need to understand fasciculation intensity and distribution. Motor unit MRI (MUMRI) can assess fasciculations in large volumes but is not widely applied. We hypothesize that a more common MRI technique, diffusion tensor imaging (DTI), can also detect fasciculation when correcting for low signal‐to‐noise ratios and signal variability. We first systematically compared MUMRI and DTI in upper leg muscles of healthy subjects (*n* = 5). Secondly, we retrospectively determined fasciculation intensity and distribution in lower extremity muscles of 30 healthy subjects using DTI (*n* = 30). DTI and MUMRI had comparable sensitivity (75%) and precision (80%) to expert reviews. In our healthy cohort, fasciculations were more prevalent in the lower legs than upper legs (13.9 ± 11.5% vs. 9.8 ± 6.3%, *p* = 0.011), particularly in the soleus (9.3 ± 8.1%). This effect persisted after normalizing for muscle volume (7.2 ± 5.1%/dm^3^ vs. 2.9 ± 1.8%/dm^3^, *p* < 0.001). Lower leg fasciculations were larger compared to upper leg fasciculations (0.81 ± 0.31 cm^3^ vs. 0.54 ± 0.15 cm^3^, *p* < 0.001). Longitudinal analysis showed consistent fasciculation distribution over 8 months (*n* = 13, ICC = 0.803). In conclusion, muscle DTI detects fasciculations in all lower extremity muscles, enabling retrospective analysis of existing datasets and reducing the need for prospective MUMRI studies if muscle DTI is already acquired.

## 1 INTRODUCTION

Fasciculations are random muscle twitches due to the spontaneous activity of motor units. The widespread presence of fasciculations is considered a characteristic of motor neuron disease (MND) (de Carvalho & Swash, 2013). Fasciculations have therefore recently been included in the consensus diagnostic criteria for MND (Shefner et al., 2020). Furthermore, fasciculations are believed to have prognostic value in MND. Nevertheless, fasciculations are also commonly reported in healthy subjects (Leite et al., 2014; Reed & Kurland, 1963; Simon & Kiernan, 2013). Therefore, to distinguish normal fasciculation prevalence from pathological fasciculations, it is important to understand the intensity and distribution of fasciculation in healthy muscles.

Traditionally needle electromyography (EMG) and more recently muscle ultrasound and surface EMG have been used to help detect fasciculation presence and distribution. EMG and ultrasound studies showed that sporadic fasciculations are not uncommon in healthy subjects and can be detected in at least one muscle, both distal and proximal (Abraham et al., 2020; Reimers et al., 1996; Sleutjes et al., 2016; Tamborska et al., 2020; Van der Heijden et al., 1994; Wenzel et al., 1998). Needle EMG, however, is invasive and painful and has a sample volume of only a few mm^3^, so false negatives are not uncommon. Ultrasound and surface EMG are a noninvasive alternative but still have a limited sample volume.

It has been shown that MRI also allows measuring fasciculations when using a pulsed‐gradient spin echo (PGSE) sequence, a technique called motor unit MRI (MUMRI) (Heskamp, Birkbeck, Baxter‐Beard, et al., 2024; Steidle & Schick, 2015; Whittaker et al., 2019). MRI has the advantage of a larger pick‐up area within a muscle compared to ultrasound, surface EMG and needle EMG. Furthermore, MRI can capture the fasciculation distribution over a large number of muscles within a single acquisition noninvasively. PGSE images are sensitive to motion and are acquired dynamically over time. Muscle twitching, involving contracting muscle fibers (e.g., fasciculations), appears as short‐living signal voids in those images. The sensitivity of the PGSE sequence to fasciculation can be set with the *b*‐value. A higher *b*‐value means a higher motion sensitivity but results in images with lower signal‐to‐noise ratios (SNR). Most MUMRI studies use a fixed *b*‐value of approximately 100–200 s/mm^2^, with a fixed gradient direction to balance motion sensitivity and SNR to detect fasciculations (Heskamp, Birkbeck, Baxter‐Beard, et al., 2024; Heskamp, Birkbeck, Hall, et al., 2024; Steidle & Schick, 2015; Whittaker et al., 2019). This results in images with a constant contrast and signal amplitude, optimized to detect the short‐living signal voids induced by fasciculation. So far only small studies using MUMRI have been performed (Heskamp, Birkbeck, Hall, et al., 2024; Schwartz, Martirosian, et al., 2018; Steidle & Schick, 2015; Whittaker et al., 2019).

Although the PGSE sequence allows fasciculation detection, the sequence is mainly used for diffusion tensor imaging (DTI) to quantitatively assess the muscle microstructure parameters, such as mean diffusivity (MD) and fractional anisotropy (FA). Not surprisingly, fasciculations have been observed in muscle DTI images (Karampinos et al., 2012; Lemberskiy et al., 2014; Otto, 2022; Schwartz, Martirosian, et al., 2018). Given that several large cohorts of muscle DTI exist and DTI is more commonly performed than MUMRI, it would be advantageous to use DTI to determine fasciculation intensity and distribution in healthy individuals instead of conducting a large prospective study with dedicated MUMRI. Importantly future studies would be able to simultaneous assess presence and distribution of fasciculation and evaluate muscle microstructure providing additional insight into muscle physiology. DTI, however, typically uses a range of *b*‐values (0–600 s/mm^2^) with varying directions, instead of an optimized fixed *b*‐value and direction. This results in a time series of images that vary in contrast and signal amplitude and have lower SNR compared to MUMRI. Despite these differences, we hypothesize that optimized data analysis methods can make DTI comparable to MUMRI for fasciculation detection. We aimed to verify this with a prospective systematic comparison of MUMRI and DTI in five healthy volunteers.

Once validated, our goal is to retrospectively determine the distribution and intensity of fasciculation in the upper and lower legs of healthy participants using an existing cohort of muscle DTI data (Forsting et al., 2022, Forsting et al., 2024; Schlaffke et al., 2024).

## 2 METHODS

The method section is divided into two parts. First, we describe the methodology for testing whether muscle DTI is comparable to MUMRI in detecting fasciculations, including imaging protocols and detection methods, in five healthy volunteers. Second, we outline the retrospective analysis of fasciculation distribution and intensity in a healthy cohort (*n* = 30) with repeated measures in 13/30.

### 2.1 DTI versus MUMRI

#### 2.1.1 Participants

We recruited five healthy participants with an age of 37 ± 5 years (range: 28–42 years; 4 male). Exclusion criteria were a history of neuromuscular disease and contra‐indications to MRI scanning. The study was approved by the Medical Ethics Committee UMC Utrecht and all participants gave written informed consent before enrolment.

#### 2.1.2 Data acquisition

The upper legs of each participant were scanned with a 3T MR scanner (Philips, Medical Systems, Best, the Netherlands) using a multielement anterior–posterior coil. Fasciculations were imaged with two PGSE sequences: the MUMRI sequence with a fixed *b*‐value and the DTI sequence with a variable *b*‐value as typically used in muscle DTI. Both sequences had a field of view of 480 × 276 mm^2^, in‐plane resolution of 3 × 3 mm^2^ and included 33 slices (thickness/gap = 6/0 mm). The fat signal was suppressed with slice selective gradient reversal and SPAIR, and a SENSE factor of 2.4 was used in the anterior–posterior direction.

For MUMRI, we acquired 45 repetitions with a *b*‐value of 200 s/mm^2^ (Δ = 18 ms), three diffusion encoding directions alternating between right–left, anterior–posterior, and feet–head, and repetition time (TR) of 3950 ms and echo time (TE) of 42 ms. For DTI, we acquired 27 repetitions with *b*‐values of 20 (3×), 50 (3×), 200 (6×), and 500 (15×) s/mm^2^, Δ = 21 ms, all with unique gradient directions, and TR of 5994 ms and TE of 50 ms. The scan times were 2 min 57 s and 2 min 46 s, respectively.

#### 2.1.3 Data analysis

Before fasciculation detection, all images were denoised using PCA denoising (Veraart et al., 2016) and registered to their first repetition using affine registration in Elastix (Klein et al., 2010; Schlaffke et al., 2019). All muscle tissue was manually delineated as a single mask. Fasciculations are best observed at higher *b*‐values (Schwartz et al., 2017), therefore only images with *b*‐values ≥200 s/mm^2^ were included in the analysis, that is, 21 repetitions for DTI (*b* = 200 and 500 s/mm^2^) and 45 repetitions for MUMRI (*b* = 200 s/mm^2^). To handle the signal variability and lower SNR observed in DTI compared to MUMRI, we employed a custom‐built detection algorithm in QMRITools (Froeling, 2019), which is described below.

For the quantification of fasciculations, we used two metrics: fasciculation detection chance and average fasciculation size. The fasciculation detection chance was defined as the number of detected fasciculations divided by the number of analyzed images times 100%. The average fasciculation size is defined as the average size of all detected fasciculations, expressed in voxels.

##### Detection algorithm

**FIGURE 1:**
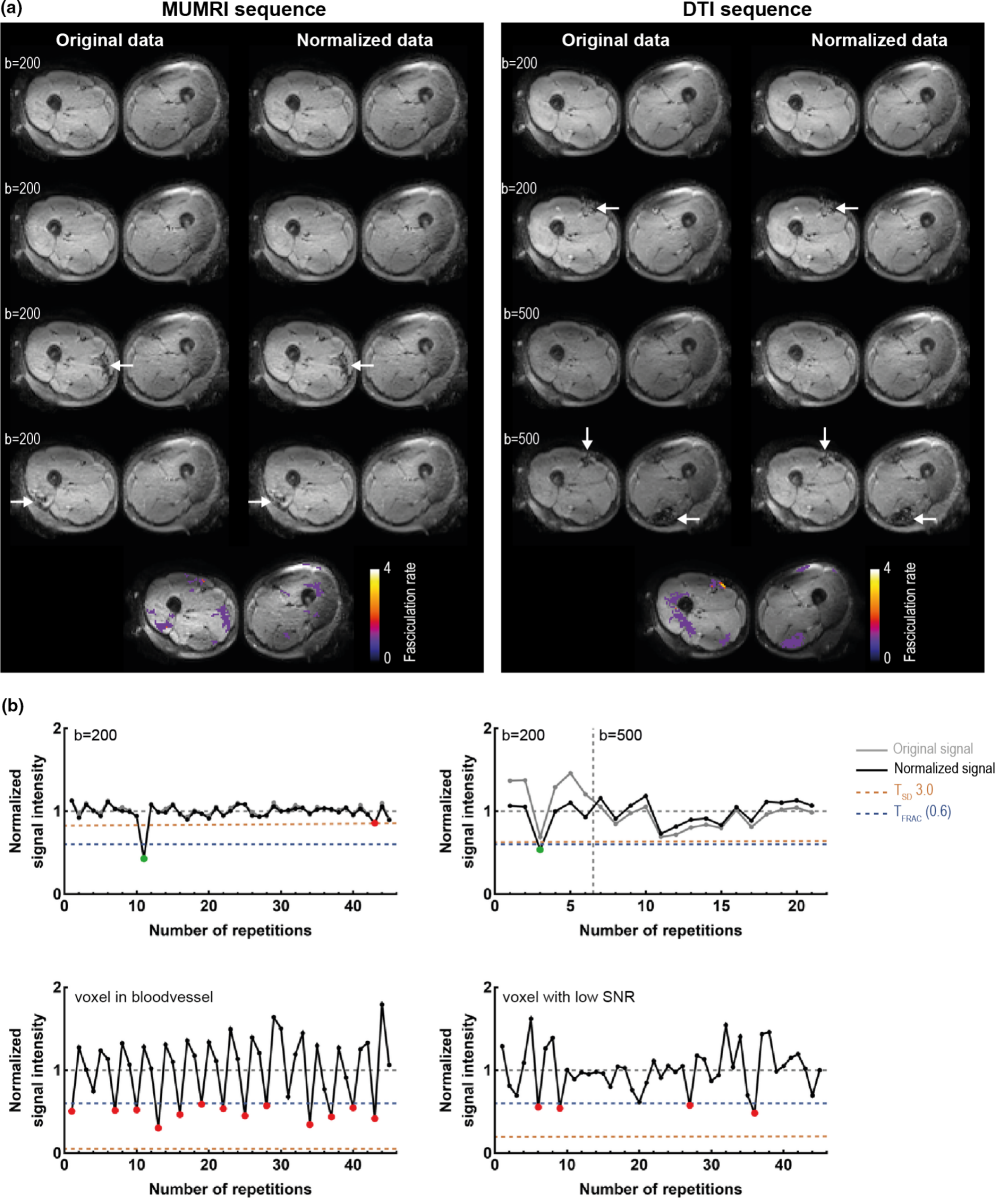
Fasciculation detection pipeline. (a) Four repetitions of MUMRI sequence at *b* = 200 s/mm^2^ and the DTI sequence (*b* = 200 and 500 s/mm^2^) pre‐normalization and post‐normalization. Sporadic signal voids are depicted with the white arrows, and the resulting fasciculation maps are displayed for *S*_FRAC_ = 0.6 and *S*_SD_ = 3.0. (b) Four signal intensity profiles pre‐normalization (gray) and post‐normalization (black). The two thresholds (*T*_FRAC_ [dashed blue] and *T*_SD_ [dashed orange]) are displayed for scaling factor 0.6 (*S*_FRAC_) and 3.0 (*S*_SD_), respectively. Top left shows an example of MUMRI data with one true fasciculation (green dot), where *T*_SD_ would have selected also one false fasciculation (red dot), but *T*_FRAC_ avoids this. Top right shows an example of DTI data where a true fasciculation would have been missed in the DTI data without normalization (green dot). The two bottom figures show a pulsating blood vessel (left) and low SNR voxel (right) where *T*_FRAC_ would have detected many false positives (red dots), but *T*_SD_ avoids this.

In the algorithm, each image was first normalized to the average signal intensity in the segmented muscle tissue to remove the signal variability induced by different *b*‐values (Figure 1a,b). Fasciculations present themselves as transient signal voids in muscle tissue. They were detected using two iteratively alternating thresholds (*T*_FRAC_ and *T*_SD_) which are calculated using:

$$T_{\text{FRAC}}=\text{mean}\;\left(\text{signa}l^{\prime }\right)\times S_{\text{FRAC}}$$

$$T_{\mathrm{SD}}=\text{mean}\;\left(\text{signa}l^{\prime }\right)-\mathrm{std}\;\left(\text{signa}l^{\prime }\right)\times S_{\mathrm{SD}}$$

With *S*_FRAC_ and *S*_SD_ being scaling factors defined by the user, and ${\text{signal}}^{\prime }$ being the signal over time including only time points with values above *T*_FRAC_ or *T*_SD_. The ${\text{signal}}^{\prime }$ is used to avoid overestimating the standard deviation in situations with high fasciculation rates.

The algorithm initialises by selecting only those time points with a value higher than *T*_FRAC_, and calculates *T*_SD_ and *T*_FRAC_ using only those time points. Thereafter, the algorithm alternates between two steps until the number of detected fasciculations stabilizes. Step 1 marks the time points with signal < Min (*T*_FRAC_, *T*_SD_) as fasciculations. Step 2 recalculates *T*_SD_ and *T*_FRAC_ using time points with signal > Min (*T*_FRAC_, *T*_SD_). For stable signals, a high *T*_FRAC_ captures all fasciculations (Figure 1b), but for variable signals due to changing diffusion contrast or blood vessel pulsation, the threshold needs to be lowered to prevent false positives. This adjustment is facilitated by *T*_SD_, which drops below *T*_FRAC_ for variable signals, lowering the threshold (see Figure 1b and Figure S1). A signal void was only classified as a fasciculation if it consisted of at least four connecting voxels to minimize the detection of noise.

##### Validation and optimization

The method is validated with Monte‐Carlo simulations detailed in the Appendix S1. We estimated the precision and sensitivity using a range of scaling factors for *T*_FRAC_ and *T*_SD_ (*S*_FRAC_: 0.1–0.9, step 0.05; *S*_SD_: 0–7, step 0.25) in simulated voxels representing no fasciculation, low to extremely high single voxel fasciculation rates (1%–50%), or a blood vessel. This simulation was performed for three SNR levels of 5, 10, and 20, with SNR 20 being the SNR expected for the MUMRI fixed b‐value of 200 s/mm^2^, and SNR 5 being the lowest SNR expected in the highest DTI *b*‐value of *b* = 500 s/mm^2^.

To determine the optimal values for *S*_FRAC_ and *S*_SD_, we determined the fasciculation detection chance and average fasciculation size in the five healthy control datasets for a comparable range of scaling factors (*S*_FRAC_: 0.1–0.9, step 0.1; *S*_SD_: 1–7, step 0.5). Because fasciculation is a stochastic process, the DTI and MUMRI datasets will contain different fasciculations wherefore one‐to‐one comparison of fasciculations is not possible. Therefore, instead, we determined the bias in fasciculation chance and size between both sequences (MUMRI minus DTI).

Thereafter, we evaluated for three sets of thresholds (<1% bias, 1% bias, and 5% bias) whether the detected fasciculations in the DTI and MUMRI datasets were indeed true fasciculations. As a reference standard, two muscle MRI experts (>10 years of experience) scored each detected fasciculation in a subset of images (3 slices equally spread along the leg [slice 10, 15, and 20] and 21 repetitions) of all DTI and MUMRI datasets as either true positive or false positive, and visually inspected the images for fasciculations missed by the detection algorithm (false negatives). Precision and sensitivity were calculated for each observer and each threshold combination.

### 2.2 Fasciculation distribution in healthy controls

#### 2.2.1 Subjects and data acquisition

Fasciculations were retrospectively examined in DTI images of the lower extremity muscles of 30 healthy controls (13 males; average age: 43 ± 14 years [range: 22–55 years]) (Forsting et al., 2022; Forsting et al., 2024; Schlaffke et al., 2024). The study was approved by the medical ethics committee of the Ruhr‐University Bochum (ethics number: 15‐5281). All participants gave written informed consent before enrolment.

The inclusion criteria for the study specified that participants should have no history of muscle or nerve diseases and no leg muscle injuries within the 12 months preceding enrolment. For follow‐up measures, participants were required to report injuries, changes in physical activity beyond routine daily activities or recreational training, or weight fluctuations of several kilograms. They were permitted to maintain their usual lifestyle without restrictions, except for avoiding exercise on the day of the MRI session. The physical activity levels of the participants ranged from low to moderate, encompassing regular moderate recreational training but excluding high‐performance sports.

Participants were scanned with a 3 T MRI scanner (Achieva X, Philips, Best, The Netherlands) and multielement anterior–posterior coil. The DTI sequence had a resolution of 3 × 3 × 6 mm^3^ and included 42 repetitions (*b*‐values = 0 (1×), 1 (6×), 10 (3×), 25 (3×), 100 (3×), 200 (6×), 400 (8×), and 600 (12×) s/mm^2^, Δ = 24 ms, TR/TE = 5000/50 ms). Anatomical images were acquired with a 4pt‐Dixon sequence (TR/TE: 210/2.6, 3.36, 4.12, 4.88 ms, FA: 8°, SENSE: 2, resolution: 1.5 × 1.5 × 6 mm^3^). Both sequences had 25 slices with a FOV of 480 × 276 mm^2^. The upper legs were imaged with two stacks (5 slices overlap) and the lower legs with one stack. A subset of subjects (*n* = 13) was scanned three times, with ~4 months in between scans to assess the physiological variability of fasciculation (Forsting et al., 2024).

#### 2.2.2 Data analysis

Eight upper leg and seven lower leg muscles were manually and bilaterally segmented on the anatomical Dixon water images using ITK‐SNAP (Yushkevich et al., 2006). To warp the Dixon‐based segmentation to the DTI data we used non‐rigid b‐spline registration which covers the warping that might be present in the DWI data. The segmented upper legs were the vastus lateralis (VL), vastus medialis (VM), rectus femoris (RF), semimembranosus (SM), semitendinosus (ST), biceps femoris (BF), sartorius (S), and gracilis (G). The segmented lower leg muscles were the tibialis anterior (TA), tibialis posterior (TP), extensor digitorum longus (EDL), peroneus (PER), soleus (SOL), gastrocnemius medialis (GM), and gastrocnemius lateralis (GL).

Fasciculations were detected in the segmented muscles of the images with *b*‐values of 200, 400, and 600 s/mm^2^ using our developed data analysis method and the optimal threshold scaling factors determined from the DTI to MUMRI comparison. For each subject and visit, fasciculation detection chance (%), fasciculation detection chance normalized to sampled muscle volume (%/dm^3^), and average fasciculation size (cm^3^) were calculated using the whole muscle compartment mask and for each muscle separately.

#### 2.2.3 Statistics

Statistical analysis was performed with Graphpad Prism 10 (GraphPad Software, Boston, Massachusetts, USA), IBM SPSS Statistics for Windows (version 25.0, Armonk, NY, USA) and Matlab (version R2021a, MathWorks Natick, MA, USA). For the cross‐sectional analysis, the (normalized) fasciculation detection chance and the average fasciculation size in the upper and lower legs were compared using a two‐sided paired *t*‐test. For the longitudinal analysis, the intraclass correlation coefficient was calculated based on mean rating (*k* = 3), absolute agreement and 2‐way mixed effects models (Koo & Li, 2016). Furthermore, the variation over time in the normalized fasciculation detection chance was evaluated according to the method of Jones et al. (2011) and Christensen et al. (2020). Significance was set at *p* < 0.05. Data are presented as mean ± SD unless otherwise stated.

## 3 RESULTS

### 3.1 DTI versus MUMRI

The normalization procedure results in a stable signal over time in the DTI signal (Figure 1b). The DTI signal is, as expected, more variable than the dedicated MUMRI signal. Videos of the original and normalized DTI and MUMRI datasets can be found in the supplemental materials (Video S1a–d). Both acquisition methods allow for the detection of fasciculation using the same data analysis method in all five subjects.

#### 3.1.1 Effect of threshold settings on fasciculation detection chance

**FIGURE 2:**
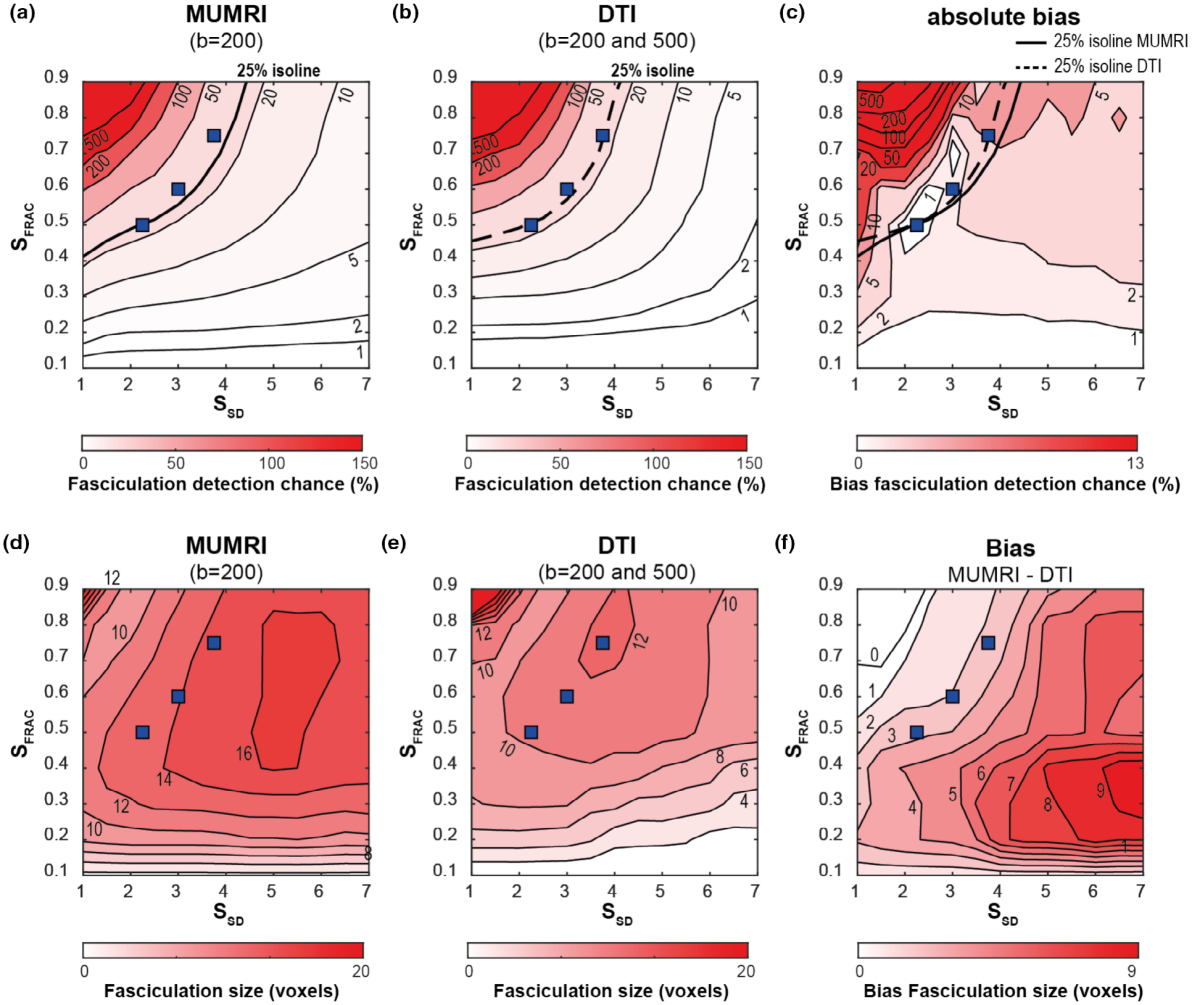
The effect of the threshold scaling factors (*S*_FRAC_ and *S*_SD_) on the fasciculation detection chance and fasciculation size. (a–c) Fasciculation detection chance for motor unit MRI (MUMRI) (a), diffusion tensor imaging (DTI) (b), and the absolute bias between MUMRI and DTI (c). The solid and dashed lines reflect the 25% fasciculation detection chance isoline for MUMRI and DTI, respectively. The three blue squares are the three scaling factor combinations for the bias levels <1%, 1%, and 5%. (d–f) Fasciculation size for MUMRI (d), DTI (e), and the bias between MUMRI and DTI (MUMRI—DTI) (c). *S*_FRAC_ and *S*_SD_ are scaling factors reflecting a fraction of the mean signal over time and a certain number of standard deviations below the mean signal over time, respectively.

After fasciculation detection, both acquisition methods show a similar dependency on the chosen threshold scaling factor, that is, the detection chance is largest for high *S*_FRAC_ and low *S*_SD_ (Figure 2a,b). The minimal absolute bias (<1%) between MUMRI and DTI is observed for *S*_FRAC_ = 0.5 and *S*_SD_ = 2.25 (Figure 2c). The corresponding fasciculation detection chance at this setting is 25%, in line with the fasciculation detection chances reported for lower leg muscles in literature (Heskamp, Birkbeck, Baxter‐Beard, et al., 2024; Heskamp, Birkbeck, Hall, et al., 2024; Schwartz, Martirosian, et al., 2018; Schwartz, Steidle, et al., 2018; Steidle & Schick, 2015).

#### 3.1.2 Effect of threshold settings on fasciculation size

The fasciculation size is largest with high *S*_FRAC_ and low *S*_SD_ for both DTI and MUMRI (Figure 2d,e), but there is also a localized maximum fasciculation size of 16 voxels (1.4 cm^2^) for MUMRI and 12 voxels (1.1 cm^2^) for DTI. The fasciculation size is on average larger for MUMRI, this bias increases with lower *S*_FRAC_ and higher *S*_SD_ (Figure 2f).

#### 3.1.3 Threshold optimization

A comparable fasciculation detection chance between MUMRI and DTI, that is, a minimal bias, does not necessarily mean that detected fasciculations are true fasciculations. Therefore, the number of false positives, precision and sensitivity were evaluated using Monte‐Carlo simulations and expert visual scoring of the acquired MUMRI and DTI images. We compared three combinations of *S*_FRAC_ and *S*_SD_ with different bias levels: minimal bias (*S*_FRAC_ = 0.5 and *S*_SD_ = 2.25), 1% bias (S_FRAC_ = 0.6 and *S*_SD_ = 3.0), and 5% bias (*S*_FRAC_ = 0.75 and *S*_SD_ = 3.75), each close to the 25% fasciculation detection chance isoline. The 25% corresponds with the fasciculation detection chances reported in previous MUMRI studies (Heskamp, Birkbeck, Baxter‐Beard, et al., 2024; Heskamp, Birkbeck, Hall, et al., 2024; Schwartz, Martirosian, et al., 2018; Schwartz, Steidle, et al., 2018; Steidle & Schick, 2015). Additionally, these values show a comparable bias in the fasciculation size but show variation in the bias for the fasciculation detection chance. When using *b*‐values of 20 s/mm^2^ and higher instead of *b*‐values of 200 s/mm^2^ or higher for DTI, similar results are obtained (Figure S2).

According to the Monte‐Carlo simulations, *S*_FRAC_ = 0.5 and *S*_SD_ = 2.25 tends to detect blood vessels as false positives even at high SNR levels and sensitivity is too low at good SNR values, wherefore it is not a suitable threshold combination, despite having the lowest bias (Figure S2). Furthermore, combination *S*_FRAC_ = 0.75 and *S*_SD_ = 3.75 significantly loses sensitivity at low SNR levels (40% at SNR 5, while >80% for SNR 10 and 20) (Figures S3–S7). Therefore, based on the simulation, *S*_FRAC_ = 0.6 & *S*_SD_ = 3.0 is the optimal choice, as it results in a precision of >70% for each SNR level while maintaining acceptable sensitivity at lower SNR (>60% at SNR 5) (Figures S3–S7). At optimal threshold combination, the algorithm is stable up to single voxel fasciculation rates of ~25% with SNR > 10 (Figure S8). Above those fasciculation rates, the algorithm will fail (Figure S9).

In the experimental data, all three threshold combinations gave comparable sensitivity and precision for both MUMRI and DTI. By applying the threshold combination of *S*_FRAC_ = 0.6 and *S*_SD_ = 3.0 to the combined DTI and MUMRI data, we achieved a precision of 80% and a sensitivity of 75% (Table 1; for interrater variability see Figure S10). Therefore, the threshold scaling factors *S*_FRAC_ = 0.6 and *S*_SD_ = 3.0 were used for subsequent analysis of the large cohort of healthy controls. For this scaling factor combination, the bias in fasciculation detection size is 2 voxels (18 mm^2^), this is 17% of the average detected fasciculation size for DTI.

**TABLE 1:** Precision and sensitivity of the optimized fasciculation detection method for motor unit MRI (MUMRI) and diffusion tensor imaging (DTI) separately and combined for the three investigated threshold scaling factor combinations (*S*_FRAC_ and *S*_SD_).

	Threshold scaling factors		Precision	Sensitivity
	*S*_FRAC_	*S*_SD_		
MUMRI	0.5	2.25	0.76	0.82
	0.6	3.0	0.80	0.80
	0.75	3.75	0.75	0.89
DTI	0.5	2.25	0.83	0.75
	0.6	3.0	0.80	0.72
	0.75	3.75	0.75	0.79
Combined	0.5	2.25	0.79	0.79
	0.6	3.0	0.80	0.75
	0.75	3.75	0.75	0.85

### 3.2 Fasciculation distribution in healthy controls

#### 3.2.1 Fasciculation detection chance

**FIGURE 3:**
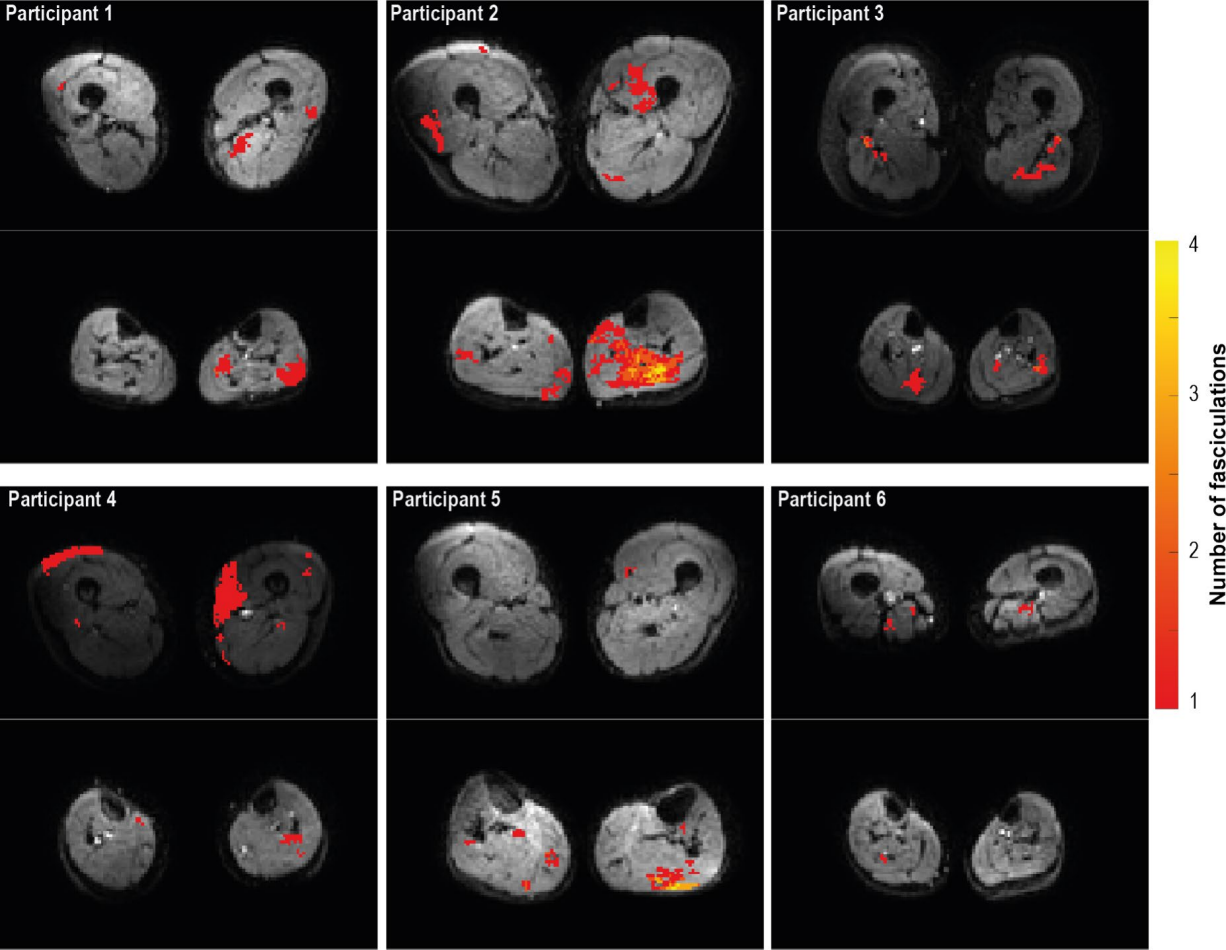
Typical fasciculation maps for the upper legs (top) and lower legs (bottom) for six healthy participants assessed with diffusion tensor imaging. Color maps display the cumulative number of fasciculations detected over all images for a single slice. The distribution of fasciculation is variable between participants, but in general, fasciculations are most prone in the posterior compartment of the lower legs.

**FIGURE 4:**
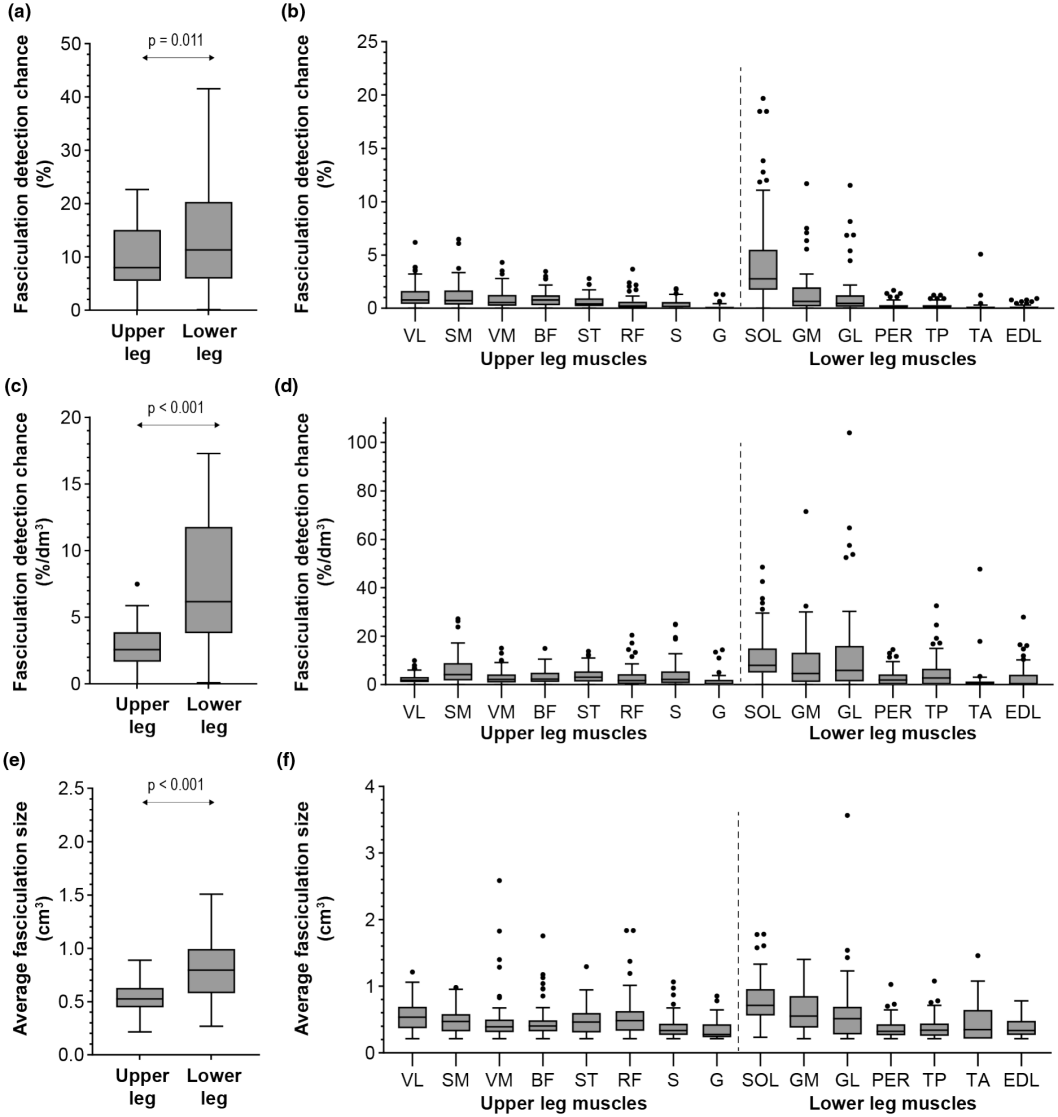
Fasciculation detection chance and fasciculation size in the lower extremity muscles of a healthy cohort (*n* = 30) detected using diffusion tensor imaging (DTI). (a, b) Fasciculation detection chance for upper leg versus lower leg muscles (a) and the individual muscles (b). The individual muscles are sorted by the average fasciculation detection chance. (c, d) Fasciculation detection chance normalized to analyzed volume (%/dm^3^) for upper leg versus lower leg muscles (c) and the individual muscles (d). (e, f) Fasciculation size for upper versus lower leg muscles (e) and individual muscles (f). For the upper leg versus lower leg comparison, a whole‐compartment mask was used including all muscles, and for the individual muscle analysis the individual muscle masks were used.

Typical examples of fasciculation maps in the upper and lower legs are displayed in Figure 3. On average the fasciculation detection chance is low, with the fasciculation detection chance being higher and more variable in the lower legs compared to the upper legs (13.9 ± 11.5% vs. 9.8 ± 6.3%, *p* = 0.011; Figure 4a). However, some participants show much higher fasciculation detection chances compared to the other participants, especially in the posterior compartment of the lower leg muscles (Figure 4b). The upper leg muscles with the on average highest fasciculation detection chances are the vastus lateralis, vastus medialis, semimembranosus, and biceps femoris (Table 2). After normalization for muscle volume, the fasciculation detection chance remains higher in the lower leg muscles compared to the upper leg muscles (7.2 ± 5.1%/dm^3^ vs. 2.9 ± 1.8%/dm^3^, *p* < 0.001; Figure 4c), with the highest fasciculation detection changes per dm^3^ observed in the lower leg's posterior compartment muscles (Figure 4d; Table 2). For the upper leg muscles, the semimembranosus has the highest fasciculation detection chance per dm^3^. When using *b*‐values of 25 s/mm^2^ and higher instead *b*‐values 200 s/mm^2^ or higher for DTI, similar fasciculation detection chances and fasciculation patterns are observed (Figure S11a,b).

**TABLE 2:** Fasciculation detection chance and fasciculation size in the lower extremity muscles of a healthy cohort detected using diffusion tensor imaging (DTI).

	Fasciculation detection chance (%)	Fasciculation detection chance per muscle volume (%/dm^3^)	Fasciculation size (cm^3^)
Upper leg muscles			
Vastus Lateralis	1.2 ± 1.2	2.6 ± 2.4	0.56 ± 0.22
Semimembranosus	1.2 ± 1.3	6.1 ± 6.3	0.49 ± 0.20
Vastus medialis	0.9 ± 0.9	3.0 ± 3.2	0.50 ± 0.42
Biceps femoris	0.9 ± 0.8	3.3 ± 3.1	0.49 ± 0.29
Semitendinosus	0.7 ± 0.6	3.8 ± 3.4	0.49 ± 0.22
Rectus femoris	0.5 ± 0.7	3.3 ± 4.4	0.57 ± 0.37
Sartorius	0.4 ± 0.4	4.3 ± 5.8	0.39 ± 0.19
Gracilis	0.1 ± 0.3	1.6 ± 2.9	0.35 ± 0.17
Lower leg muscles			
Soleus	4.6 ± 4.7	11.8 ± 10.6	0.80 ± 0.35
Gastrocnemius medialis	1.5 ± 2.2	8.8 ± 11.8	0.62 ± 0.30
Gastrocnemius lateralis	1.2 ± 2.2	12.1 ± 19.1	0.62 ± 0.55
Peroneus	0.3 ± 0.4	2.9 ± 3.6	0.38 ± 0.18
Tibialis posterior	0.2 ± 0.3	4.7 ± 6.6	0.40 ± 0.20
Tibialis anterior	0.2 ± 0.7	1.6 ± 6.5	0.46 ± 0.34
Extensor digitorum	0.1 ± 0.2	2.8 ± 5.4	0.38 ± 0.15

*Note*: *n* = 60, two muscles (right and left leg) per subject. Muscles are sorted by the average fasciculation detection chance.

#### 3.2.2 Fasciculation size

The average fasciculation size was larger in the lower leg muscles compared to the upper leg muscles (0.81 ± 0.31 cm^3^ vs. 0.54 ± 0.15 cm^3^, *p* < 0.001) (Figure 4e). This difference between the lower leg and upper leg was no longer observed within individual muscles, likely because large fasciculations in the lower leg's posterior compartment covering the soleus and gastrocnemius muscles are split into smaller signal voids (Figure 4f). The soleus is, however, still the muscle with the largest average fasciculation size followed by the gastrocnemius medialis, gastrocnemius lateralis, rectus femoris, and vastus lateralis. The on average smallest fasciculation sizes are observed in the sartorius, gracilis, extensor digitorum, and peroneus.

#### 3.2.3 Longitudinal follow‐up

**FIGURE 5:**
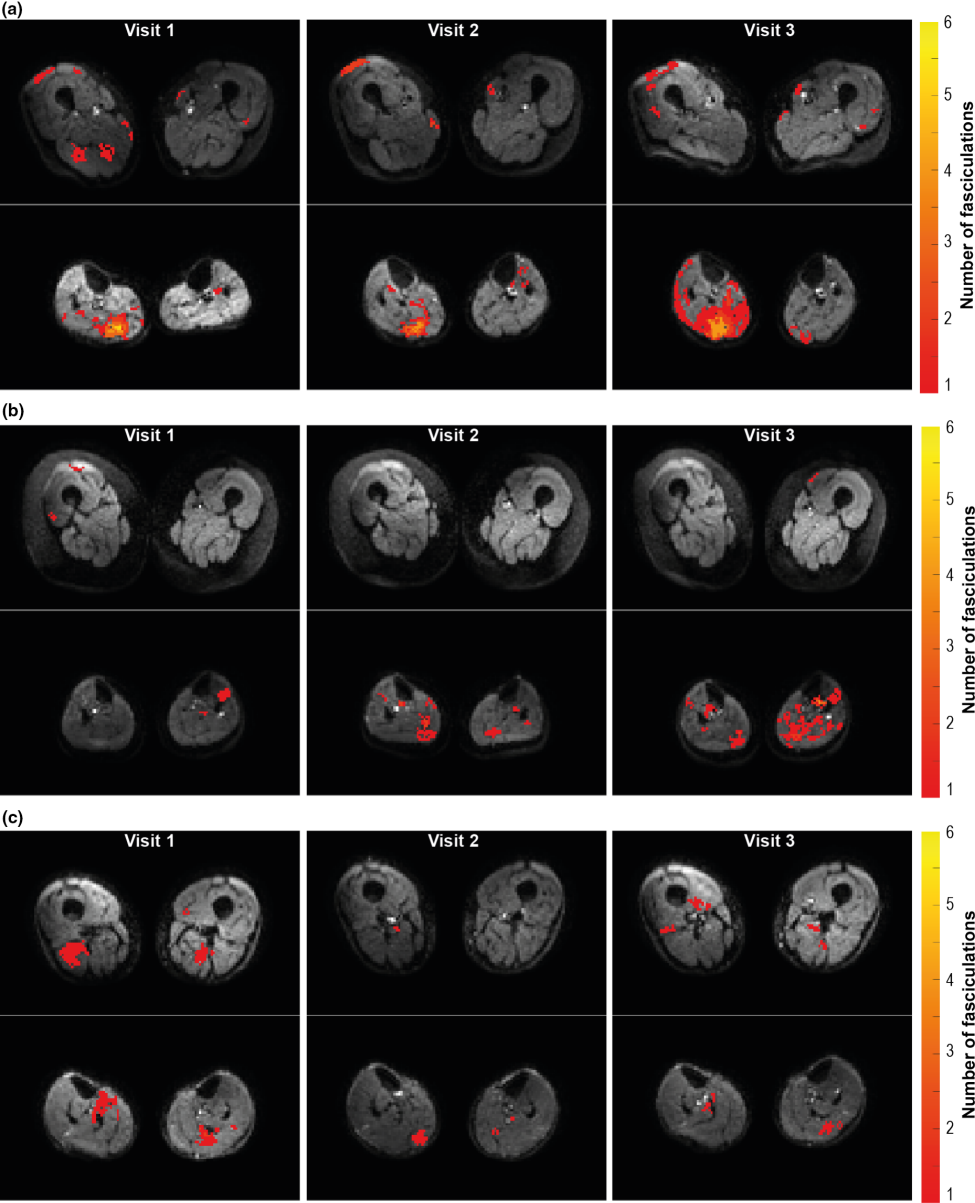
Typical fasciculation maps over three visits for the upper legs and lower legs of healthy participants assessed with diffusion tensor imaging (DTI). Color maps display the cumulative number of fasciculations detected over all images for a single slice. These fasciculation maps show that the fasciculation intensity is repeatable over time. Exceptions exist, the fasciculation intensity in the lower legs of the participant displayed in b is much lower in visit 1 compared to visits 2 and 3.

**FIGURE 6:**
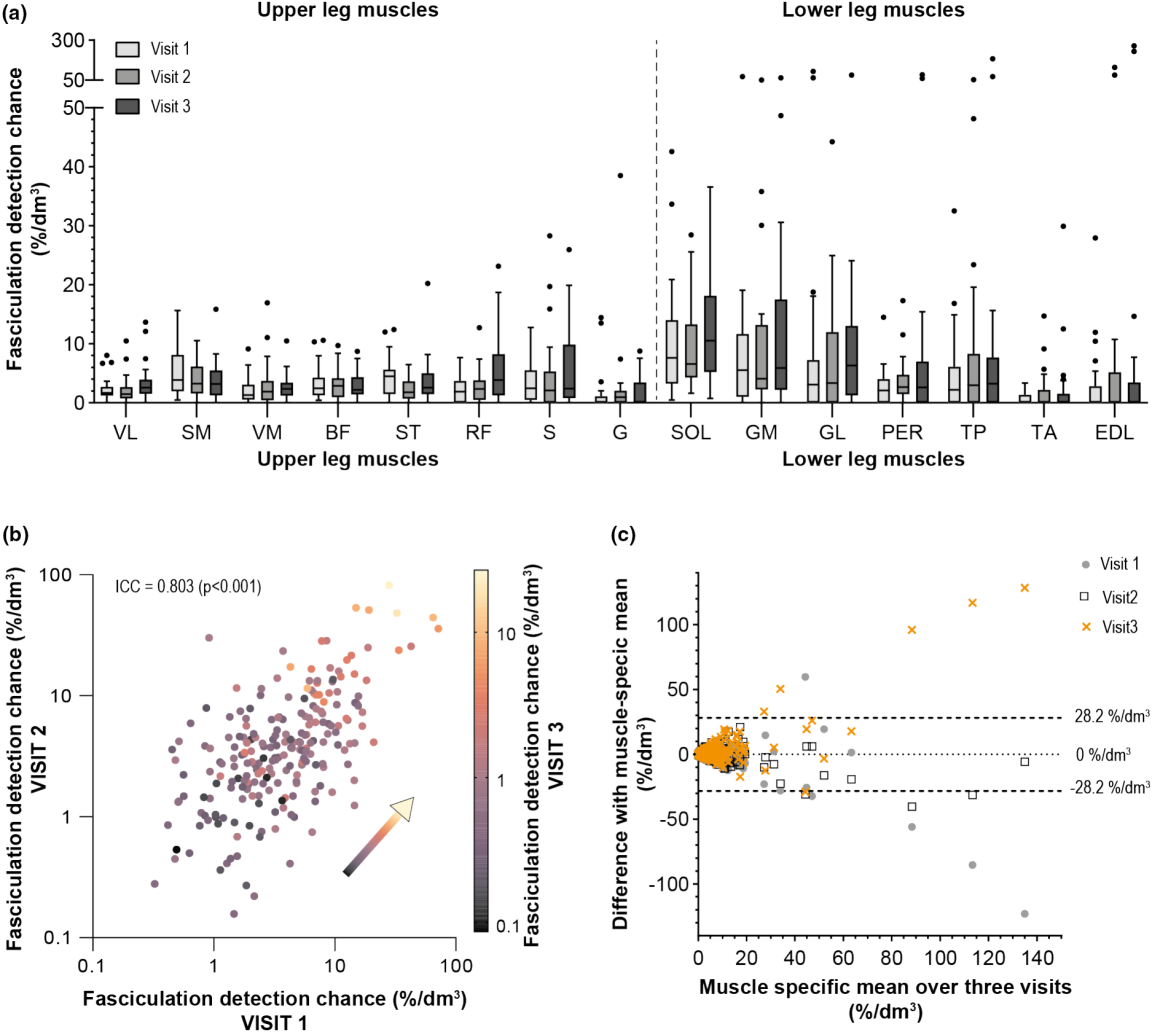
Longitudinal follow‐up of fasciculation detection chance normalized to muscle volume (%/dm^3^) in 13 healthy participants. (a) Distribution over the individual lower extremity muscles per visit. (b) Color correlation plot of visit 1 versus visit 2 versus visit 3. The arrow displays the direction of the correlation. (c) Jones plot depicting the muscle‐specific mean over the three visits against the difference between the muscle‐specific value at each visit and the muscle‐specific mean over the three visits (visit 1: Gray dots, visit 2: Black open squares, and visit 3: Orange cross). The dotted line is the bias and the dashed lines are the limits of agreements.

Of the 30 included participants, 13 participants were scanned three times to investigate the physiological repeatability of fasciculation over time. The average time in‐between visit 1 and visit 2 and visit 1 and visit 3 was 4.0 ± 0.5 months and 8.7 ± 1.5 months, respectively. The distribution of the normalized fasciculation detection chance between muscles was comparable between the three visits (Figures 5 and 6a). The intraclass coefficient for the fasciculation detection chance and normalized fasciculation detection chance were 0.826 (*p* < 0.001) and 0.803 (*p* < 0.001), respectively. (Figure 6b). In general, muscles with low fasciculation detection chances at visit 1 also show low fasciculation detection changes at visit 2 and 3, and muscles with high fasciculation detection changes at visit 1 remain high at visit 2 and 3. However, this is not always the case and the Jones plot shows that the variation over time is larger in muscles with an average higher fasciculation detection chance (Figure 6c). There was no bias and the overall limits of agreement are −28.2%/dm^3^ and 28.2%/dm^3^. When using *b*‐values of 25 s/mm^2^ and higher instead of *b*‐values of 200 s/mm^2^ or higher for DTI, a similar variability over time was observed (Figure S11c,d).

## 4 DISCUSSION

We demonstrated that image normalization combined with an iterative detection algorithm effectively identifies fasciculations as sporadic transient signal voids in DTI images. Both DTI and MUMRI exhibited similar response patterns when threshold levels were optimized. Within a specific threshold range, both techniques yielded comparable fasciculation detection chances. Moreover, both DTI and MUMRI closely matched the ground truth, as determined by visual assessments conducted by two experienced observers. Therefore, our developed method can be used to retrospectively detect the intensity and distribution of fasciculations in already acquired DTI data. This delivers information on the relative intensity and distribution of fasciculation in healthy muscle, which is needed to distinguish normal fasciculation from pathological fasciculation.

This retrospective analysis of the DTI images in a healthy cohort revealed a significantly higher fasciculation prevalence in the lower legs (13.9%) compared to the upper legs (9.8%). Within the lower legs, the posterior compartment had the highest fasciculation detection chance (9.3% in SOL vs. 0.3% in TA). In the upper legs, fasciculations were most prevalent in the semimembranosus. This fasciculation distribution is in line with previous large cohort ultrasound and EMG studies, as well as results from exploratory MUMRI studies on smaller populations (Heskamp, Birkbeck, Baxter‐Beard, et al., 2024; Heskamp, Birkbeck, Hall, et al., 2024; Reimers et al., 1996; Steidle & Schick, 2015; Van der Heijden et al., 1994; Wenzel et al., 1998). Therefore, it is recommended to focus on the proximal muscles for diagnostic purposes since high fasciculation detection chances in those muscles are uncommon and, therefore, more likely to be pathological.

The fasciculation detection chance in our healthy cohort is lower compared to the fasciculation detection chance reported in prior MUMRI studies and the five healthy subjects used for data analysis optimization; they recorded lower leg fasciculation detection chances between 16% and 25% (Heskamp, Birkbeck, Baxter‐Beard, et al., 2024; Heskamp, Birkbeck, Hall, et al., 2024; Schwartz, Martirosian, et al., 2018; Schwartz, Steidle, et al., 2018; Steidle & Schick, 2015). Additionally, the average fasciculation size in our study was 0.81 cm^2^ in the lower legs and 0.54 cm^2^ in the upper legs, ranging from 0.2 to 3.5 cm^2^. This is lower compared to previous MUMRI work, which reported an average fasciculation size of 1.5 cm^2^ in the lower legs (range ~0.1 to 8 cm^2^) (Schwartz, Martirosian, et al., 2018). These findings suggest that our method is more conservative than previous studies. This is because we designed our algorithm, that is, the iterative thresholding, to prevent the detection of false positives like blood vessels and noise in the relatively lower SNR DTI images. The consequence of this conservative approach is that some true fasciculations may be missed, for example, in cases with a coil sensitivity profile induced localized low SNR areas at the lateral sides of thighs. According to our simulations, the sensitivity is at least 60% for single‐voxel SNR values >5, except for extremely high single‐voxel fasciculation rates (>25%). Furthermore, differences may be explained by the acquisition protocol, that is, differences in the sequence's motion sensitivity level. This means that at this stage, we recommend interpreting the fasciculation data, both from our study and others, in terms of fasciculation patterns and detection chances among individual muscles, rather than relying on absolute fasciculation detection chances as reference values for normal fasciculation. For instance, our findings indicate that if one finds a significantly higher fasciculation detection chance in the lower legs' anterior compartment compared to the posterior compartment, this suggests a deviation from the normal pattern.

Longitudinal follow‐up of healthy subjects to evaluate the physiological variation of fasciculation over 8 months shows that the within‐subject fasciculation distribution pattern remains consistent over time. The exact fasciculation detection chances vary, with an average variability of ~60%. Generally, if a muscle has a high fasciculation detection chance at baseline, it will have a high fasciculation detection chance at adjoining follow‐ups, but this is not always the case. Furthermore, for muscles with high fasciculation rates, the variability in fasciculation detection chance is high. Such physiological variability has also been demonstrated in fasciculation rates using surface EMG. Healthy subjects showed major fluctuations in fasciculation rate within a single day and over 10 successive days (Van der Heijden et al., 1994). Furthermore, in ALS patients errors up to 18% were reported when comparing a shortened 5‐min recording to the full 30‐min recording (Crook‐Rumsey et al., 2022), and daytime consistency was stable in some patients, while in others fasciculation rates doubled or halved during the day (Bashford et al., 2020). (Van der Heijden et al., 1994). Our reported coefficient of repeatability is 28%/dm^3^, this equals five times the mean fasciculation detection chance. This is still twice as low as the difference observed between ALS patients and healthy controls with MUMRI, suggesting that DTI is able to detect abnormally increased levels of fasciculation.

The observed variation between subjects and over time suggests the presence of physiological provoking factors that influence fasciculations, like exercise, caffeine intake and age. The literature consistently agrees that exercise has a profound effect on fasciculations. Fasciculations were more prevalent in the gastrocnemius and abductor hallucis longus in three out of 10 subjects 30 min after strenuous exercise compared to before the exercise (Fermont et al., 2010). Another study showed that the fasciculation pattern differed between runners and handball players after exercise, with upper leg fasciculations being especially prevalent in runners and upper arm fasciculations in handball players (Czell et al., 2016). In our cohort, the amount of exercise was not reported. Participants were asked not to perform any exercise on the day of the MRI examination and none of the participants reported major changes in physical activity over the time of follow‐up examinations. The effect of caffeine intake on fasciculation prevalence could not be demonstrated in a systematic ultrasound study (Fermont et al., 2010), caffeine intake was not reported in our cohort. The effect of age on fasciculations is contradictory. Some studies show an increase in fasciculations with age (Fermont et al., 2010; Johansson et al., 2017), while others show no effect (Falck & Alaranta, 1983; Reimers et al., 1996; Wenzel et al., 1998). Furthermore, when interpreting within‐subject variation, factors like measurement duration and volume, muscle group assessed and selection of outcome measures should be considered (Crook‐Rumsey et al., 2022; Mills, 2011; Noto et al., 2017).

To enable comparison of fasciculation detection chances and sizes between studies, the underlying technical methods, particularly the detection algorithm, must also be considered. We showed that the chosen threshold scaling factors in our iterative fasciculation detection algorithm are crucial for the estimated fasciculation detection chance and size. When chosen too high or too low, fasciculation detection chances will be over‐ or under‐estimated, depending on the situation. Generally, a lower *S*_FRAC_ and higher *S*_SD_ reduce the fasciculation detection chance, but the size of this effect depends on the combination of *S*_FRAC_ and *S*_SD_. In our experiment with five healthy subjects, all three tested threshold scaling factor combinations yielded a 25% fasciculation detection chance for DTI but identified different signal voids. The combination *S*_FRAC_ = 0.5 and *S*_SD_ = 2.0 often detected false positives, like blood vessels and noisy patches. In contrast, the combination *S*_FRAC_ = 0.75 and *S*_SD_ = 3.75 missed true fasciculations and fragmented larger true fasciculations into multiple smaller ones. The combination *S*_FRAC_ = 0.6 and *S*_SD_ = 3.0 most closely matched visual fasciculation identification by two experienced observers.

Regarding fasciculation size, higher *S*_FRAC_ and lower *S*_SD_ generally correlate with larger fasciculations. In high SNR muscle tissue, lower *S*_FRAC_ results in smaller fasciculation sizes because the edge voxels fail to meet the threshold. Conversely, higher *S*_SD_ in voxels with high temporal variations, for example, noisy voxels or blood vessels, results in larger fasciculation sizes by excluding the small patches belonging to blood vessels and noise. However, extremely high *S*_SD_ values decrease fasciculation size by being overly conservative, causing fragmentation into smaller fasciculations. The effect of the chosen threshold on the reported fasciculation detection changes and sizes is not limited to our study but also applies to previous work that used specific fasciculation detection thresholds (Heskamp, Birkbeck, Hall, et al., 2024; Steidle & Schick, 2015; Whittaker et al., 2019). More research is therefore needed to determine the exact influence of threshold scaling factor choice on the fasciculation pattern and fasciculation detection chance and size.

Here we introduced a new algorithm for more robust fasciculation detection in muscle DTI data based on iterative thresholding. The main limitation is that we validated our fasciculation detection algorithm on DTI and MUMRI data on only five healthy volunteers. Nevertheless, the threshold effect on sensitivity and precision already shows the same pattern as the Monte‐Carlo simulations. Other algorithms might not suffer from the same limitations or dependencies (Schwartz et al., 2020; Schwartz, Steidle, et al., 2018).

In conclusion, our study demonstrates that DTI can reliably detect the presence and distribution of fasciculations provided that signal variations and lower SNR are properly addressed in the data analysis. This can be valuable since it allows for retrospective analysis of existing DTI datasets mitigating the need for dedicated MUMRI acquisitions if DTI is already acquired, and allowing simultaneous assessment of muscle microstructure parameters and presence and distribution of fasciculations in both retrospective and prospective studies. In this first retrospective analysis, we found that in healthy controls fasciculations are most prevalent in the lower extremity muscles and that the fasciculation pattern is stable over time, although some physiological variation is observed. As with any other quantitative fasciculation measurement technique, the exact quantification of fasciculation detection chances and sizes remains challenging as it depends on the data analysis methods. Given that fasciculation imaging of large volumes is an emerging field, fasciculation imaging with MRI can aid in the earlier diagnosis of neuromuscular disorders like ALS where large differences in fasciculation are expected. However, in case of small expected differences, single fasciculation measures might not provide the desired information due to the large subject variance, in those cases longitudinal examinations are suggested.

## FUNDING INFORMATION

This work was supported by VIDI research programme (project number: 18929) of the Dutch Research Council (NWO) and Sanofi (SGZ‐2019‐12541).

## CONFLICT OF INTEREST STATEMENT

All authors declare no competing interests.

## ETHICS STATEMENT

Study participants provided written informed consent to participate. The study was reviewed and approved by the Medical Ethics Committee UMC Utrecht and medical ethics committee of teh Ruhr‐University Bochum (ethics number: 15‐5281).

## Supporting information


Appendix S1.

Video S1.

## Data Availability

The data that support the findings of this study are available from the corresponding author upon reasonable request.
