# Supplementary material for: Fasciculation distribution in a healthy population assessed with diffusion tensor imaging
Source: Physiol Rep. 2025 Mar 22;13(6):e70247. doi: 10.14814/phy2.70247 (PMC11928744; doi:10.14814/phy2.70247)
Supplement: Supplementary file 1 — Appendix S1. [file PHY2-13-e70247-s001.docx]

# Supplementary

## Fasciculation detection thresholds

Supplemental Figure 1 displays typical example maps of the threshold values for both DTI (top) and MUMRI (bottom). The left figure shows the SD threshold (T_SD_) values for a scaling factor of 3.0 and the middle figure shows the fraction threshold (T_FRAC_) for a scaling factor of 0.6. The right figure shows the chosen threshold to detect fasciculation, *i.e.* the lowest value of the two thresholds. Two important observations can be noted. First, in most voxels, the T_FRAC_ is lower than T_SD_, and thus in general T_FRAC_ is the chosen threshold to detect fasciculation. T_SD_ becomes leading when signal variation is high, for example in noisy areas, or blood vessels. Second, the T_SD_ values are generally lower for the DTI data compared to the MUMRI data, which is due to the signal variations induced by the varying b-values. Our approach of two iteratively alternating thresholds is therefore especially important when detecting fasciculation using DTI data.


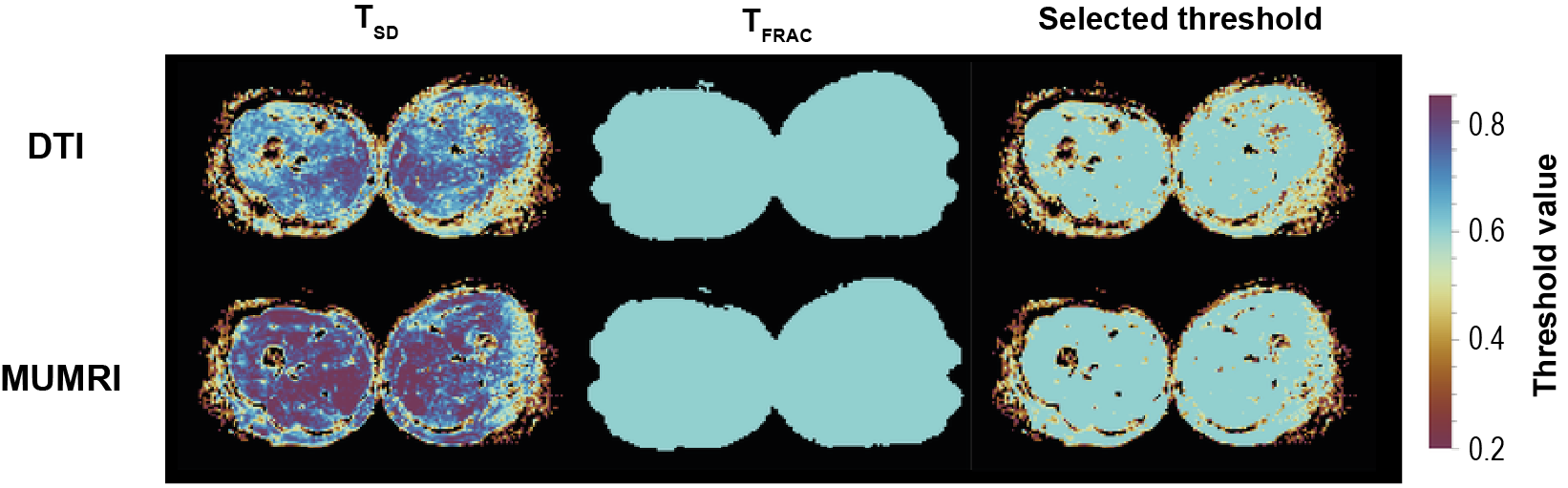


**Figure S1**

## Effect of b-value on the DTI vs MUMRI comparison

##
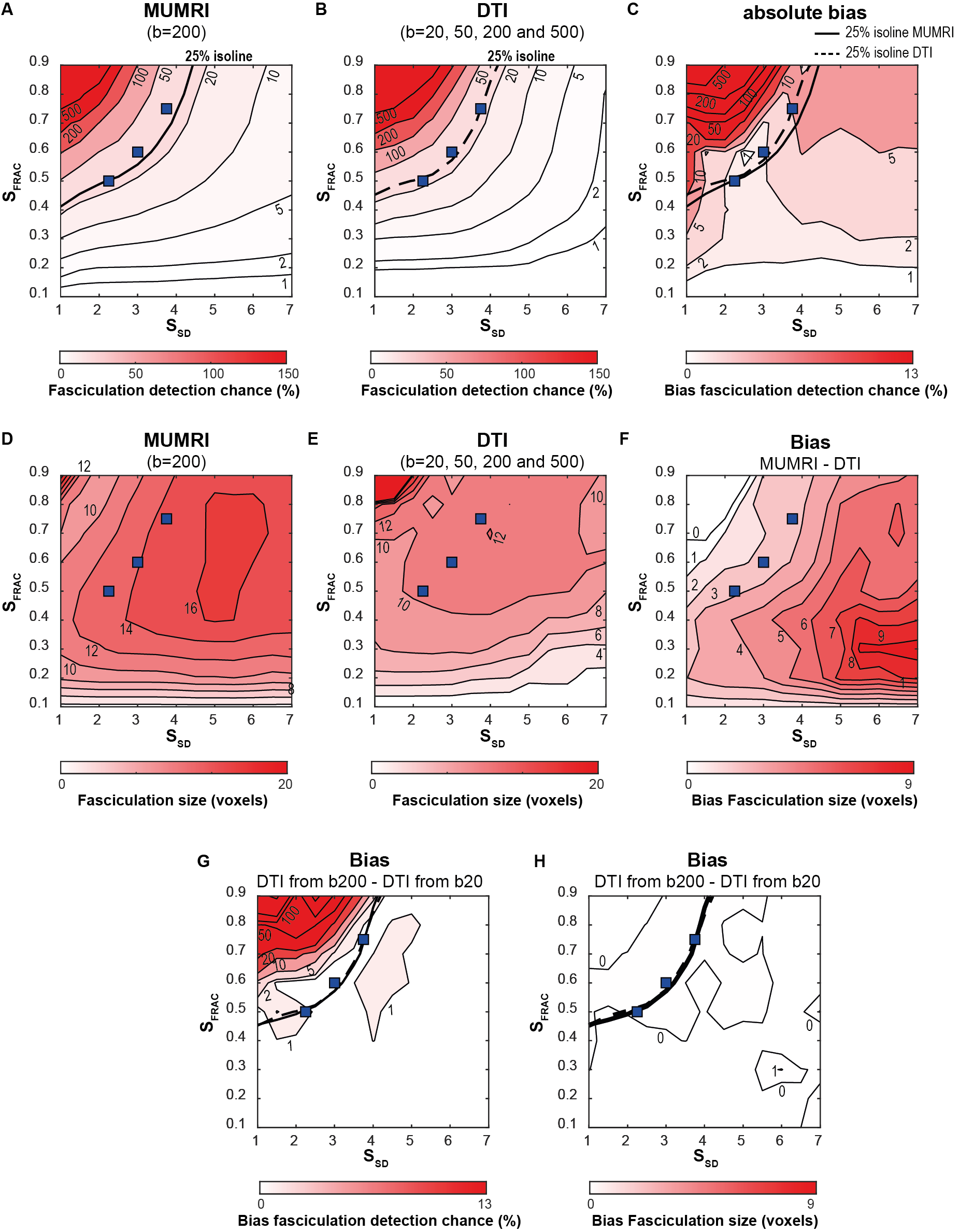


**Figure S2:** The effect of the threshold scaling factors (S_FRAC_ and S_SD_) on the fasciculation detection chance and fasciculation size for the DTI data including b-values of 20 s/mm^2^ and higher. A-C) Fasciculation detection chance for motor unit MRI (MUMRI) **(A)**, diffusion tensor imaging (DTI) with b-values of 20 s/mm^2^ or higher **(B)** and the absolute bias between MUMRI and DTI **(C)**. The solid and dashed lines reflect the 25% fasciculation detection chance isoline for MUMRI and DTI, respectively. The three blue squares are the three scaling factor combinations for the bias levels <1%, 1% and 5%. D-F) Fasciculation size for MUMRI **(D)**, DTI with b-values of 20 s/mm^2^ or higher **(E)** and the bias between MUMRI and DTI (MUMRI - DTI) **(C)**. G-H) Bias in fasciculation detection chance **(G)** and size **(H)** between DTI using b-values of 200 s/mm^2^ and higher minus DTI using b-values of 20 s/mm^2^ and higher. S_FRAC_ and S_SD_ are scaling factors reflecting a fraction of the mean signal over time and a certain number of standard deviations below the mean signal over time, respectively.

## Monte-Carlo simulations

### Methods:

For seven scenarios, we simulated time series to represent the DTI signal over time. For each scenario, 10000 time series were simulated with 20 dynamics each, i.e. 200.000 points.

1. Single voxel in blood vessels, which have a random signal, without fasciculation (0%).
2. Single voxel in muscle tissue, which has a constant signal, without fasciculation (0%).
3. Single voxel in muscle tissue with a low fasciculation rate (1%); 1 in 100 data points is a fasciculation, i.e. 200 fasciculations spread over the 10000 time-series.
4. Single voxel in muscle tissue with a medium fasciculation rate (2%); 1 in 50 data points is a fasciculation, i.e. 400 fasciculations spread over the 10000 time-series.
5. Single voxel in muscle tissue with a high fasciculation rate (5%); 1 in 20 data points is a fasciculation, i.e. 1000 fasciculations spread over the 10000 time-series.
6. Single voxel in muscle tissue with extremely high fasciculation rate (25%); 1 in 4 data points is a fasciculation, i.e. 2500 fasciculations spread over 10000 time-series.
7. Single voxel in muscle tissue with extremely high fasciculation rate (50%) where the algorithm misclassifies fasciculation as pulsation; 1 in 2 data points is a fasciculation, i.e. 5000 fasciculations spread over 10000 time-series.algorithm fails at all SNR levels (50%)

All scenarios were evaluated at three noise levels, a signal-to-noise ratio (SNR) of 5, 10 and 20. An SNR level of 5 can be expected at the highest b-values in muscle DTI, while SNR 20 is expected for MUMRI.

For each simulation, we determined the number of sensitivity, precision, as well as the estimated fasciculation rate and the average count of false positives per 20 time points.. A sensitivity and precision of 80% or higher is assumed to be acceptable.

The amount of signal drop for each fasciculation was simulated using a Rayleigh distribution, where most true fasciculations had a signal drop below 60% of the original signal. Around 10% of the fasciculations have a signal drop between 60 and 90% of the original signal.


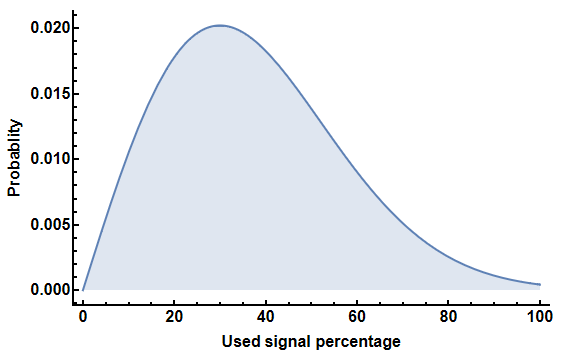


### Results

For each scenario, the results are displayed in the figures below. In these figures, the top row shows the simulated time series for the three different SNR levels (left: SNR 5, middle: SNR 10, right SNR 20). The 4 colour maps at the bottom row display the sensitivity (top left), precision (top right), detected fasciculation rate (bottom left) and number of false positives (bottom right) for each investigated threshold scaling factor combination with the standard deviation scaling factor (S_SD_) on the x-axis and the fraction scaling factor (S_FRAC_) on the y-axis.

#### Blood vessel

Since there is no fasciculation, there are no true positives and therefore sensitivity and precision are always zero.

If only S_FRAC_ is used (S_SD_ = 0), blood vessels will be falsely detected as fasciculation, regardless of the chosen S_FRAC_. This issue can be mitigated by adding S_SD_. The less conservative S_FRAC_, the more conservative S_SD_ needs to be to avoid detecting blood vessels as fasciculation.


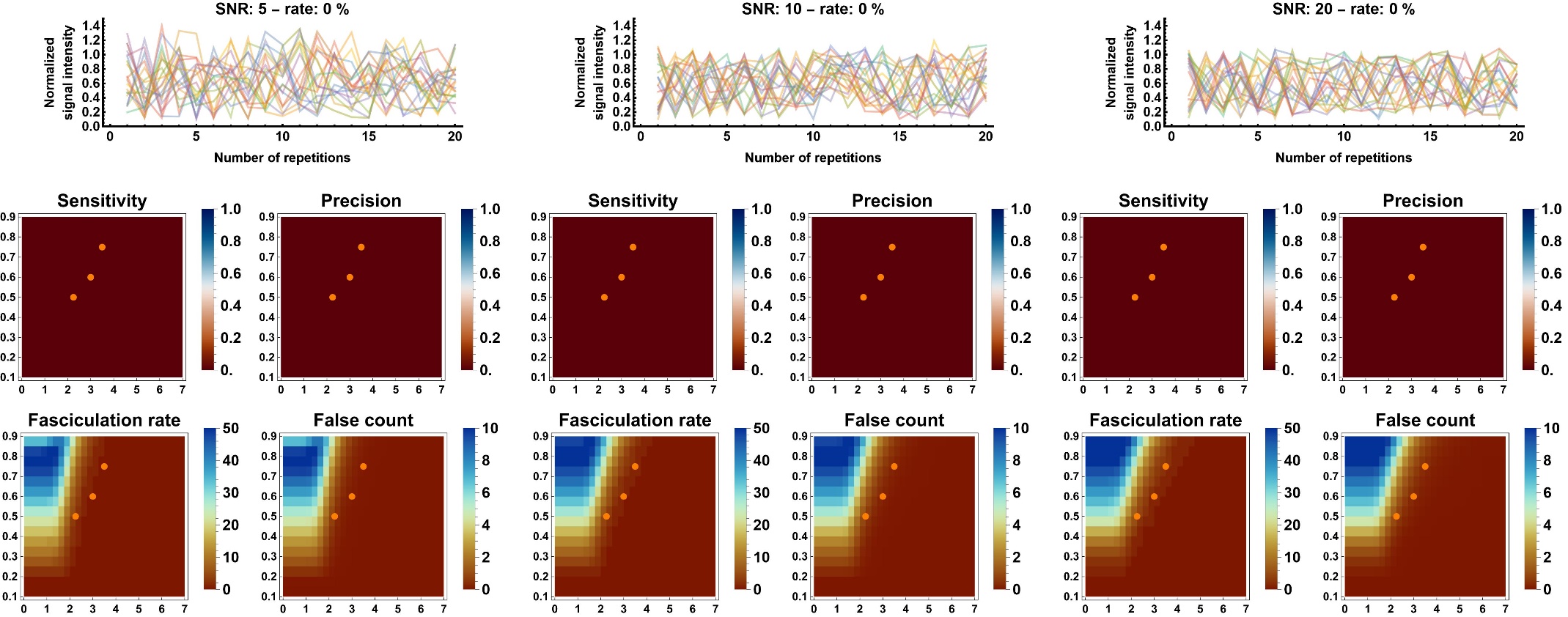


**Figure S3:** Monte-Carlo simulations for a single voxel in a blood vessel.

#### Muscle tissue without fasciculation (0%)

Since there is no fasciculation, there are no true positives and therefore sensitivity and precision are always zero.

At high SNR levels, the number of false positives is zero, and S_SD_ has no or minimal effect. At lower SNR levels, S_SD_ becomes important to avoid the detection of noise as false positives.


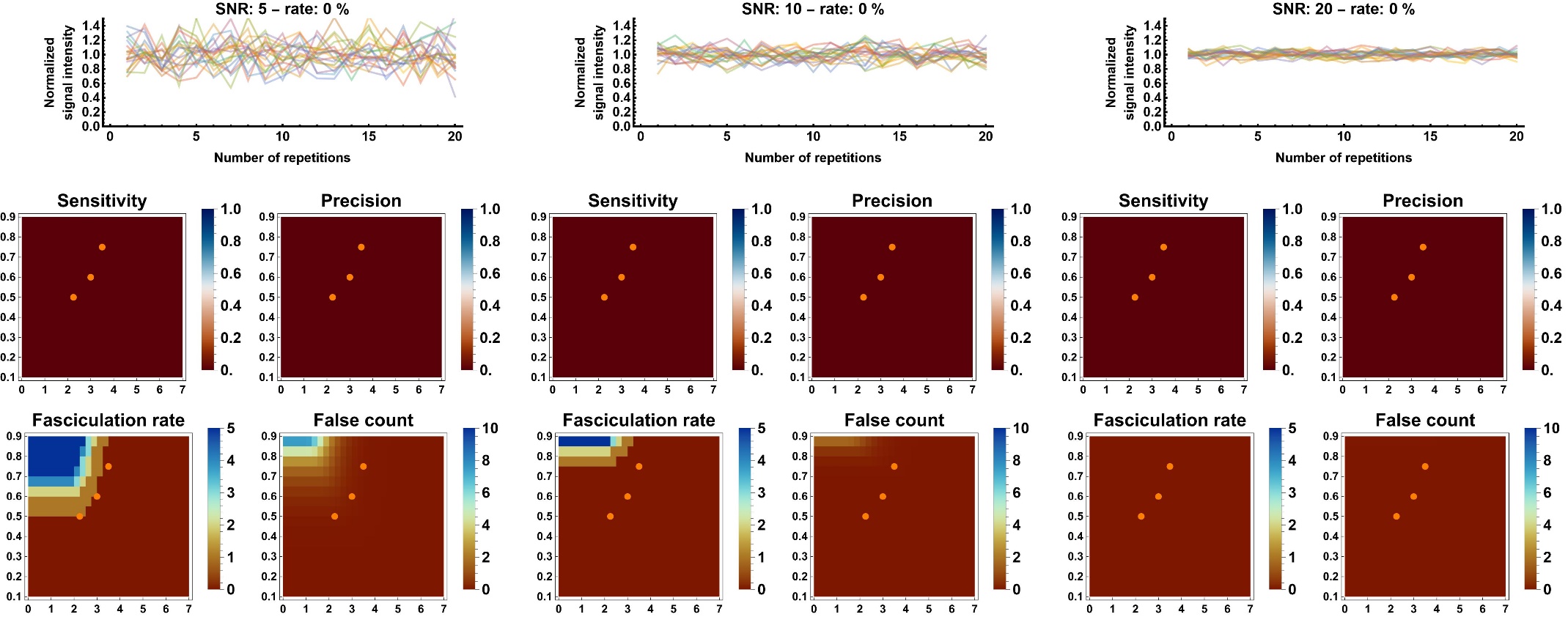


**Figure S4:** Monte-Carlo simulations for a single voxel in muscle tissue without fasciculation.

#### Muscle tissue with a low fasciculation rate (1%)

At high SNR levels, precision and sensitivity are driven by S_FRAC_, and S_SD_ has no or minimal effect. At lower SNR levels, S_SD_ becomes important to avoid the detection of noise as false positives.


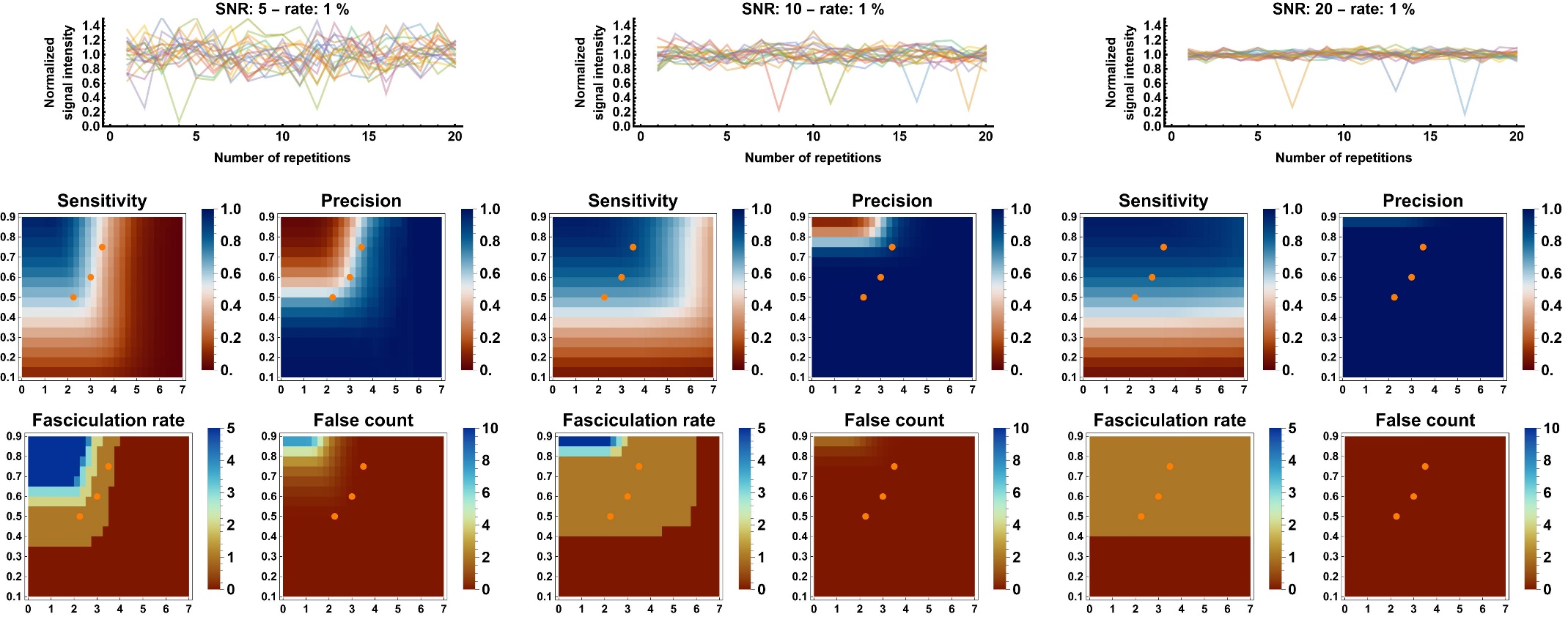


**Figure S5:** Monte-Carlo simulations for a single voxel in muscle tissue with a low fasciculation rate.

#### Muscle tissue with a medium fasciculation rate (2%)

At high SNR levels, precision and sensitivity are driven by S_FRAC_, and S_SD_ has no or minimal effect. At lower SNR levels, S_SD_ becomes important to avoid the detection of noise as false positives.


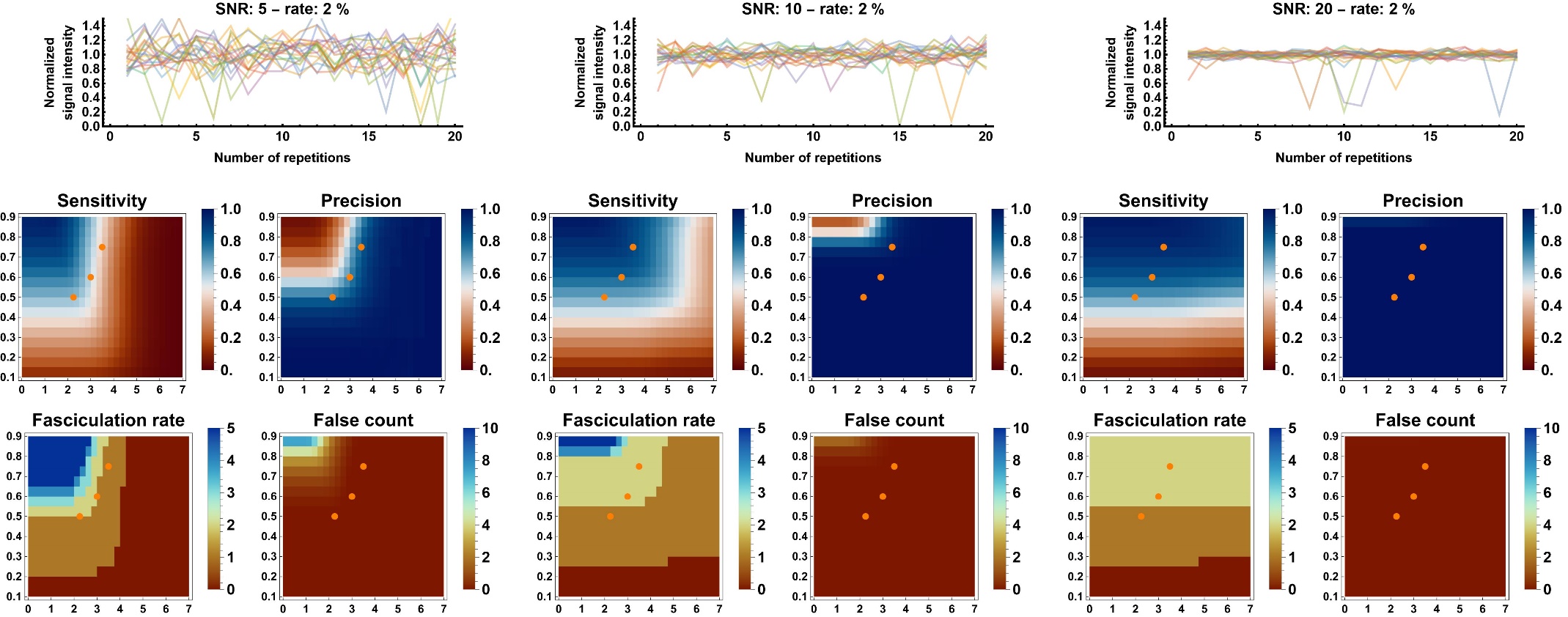


**Figure S6:** Monte-Carlo simulations for a single voxel in muscle tissue with a medium fasciculation rate.

#### Muscle tissue with a high fasciculation rate (5%)

At high SNR levels, precision and sensitivity are driven by S_FRAC_, and S_SD_ has no or minimal effect. At lower SNR levels, S_SD_ becomes important to avoid the detection of noise as false positives.


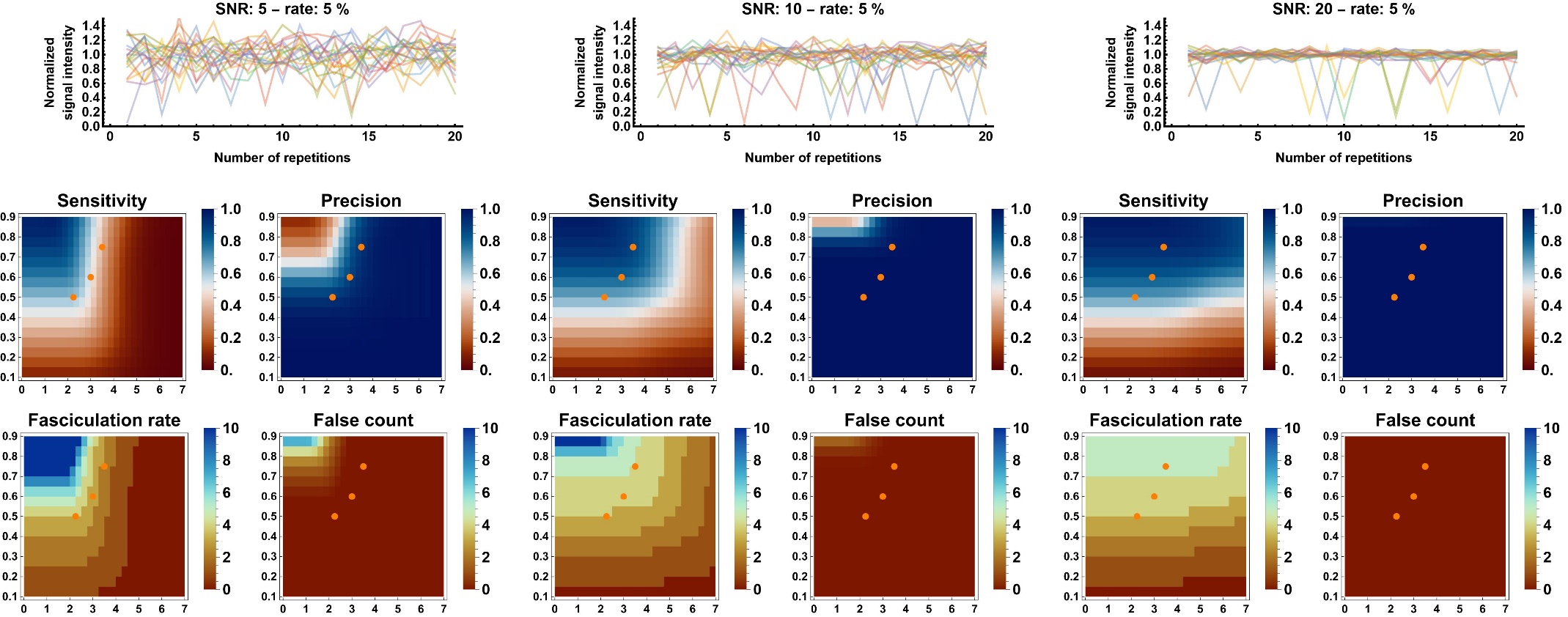


**Figure S7:** Monte-Carlo simulations for a single voxel in muscle tissue with a high fasciculation rate.

#### Muscle tissue with an extremely high fasciculation rate (25%), with algorithm still stable.

Precision is high at all SNR levels, but SNR is around 40% for all threshold combinations at an SNR level of 5. For SNR 10 and 20, sensitivity is still at 60% or above. This means that the algorithm is capable of detecting fasciculation up to a single voxel fasciculation rate of 25%, at SNR levels > 10.


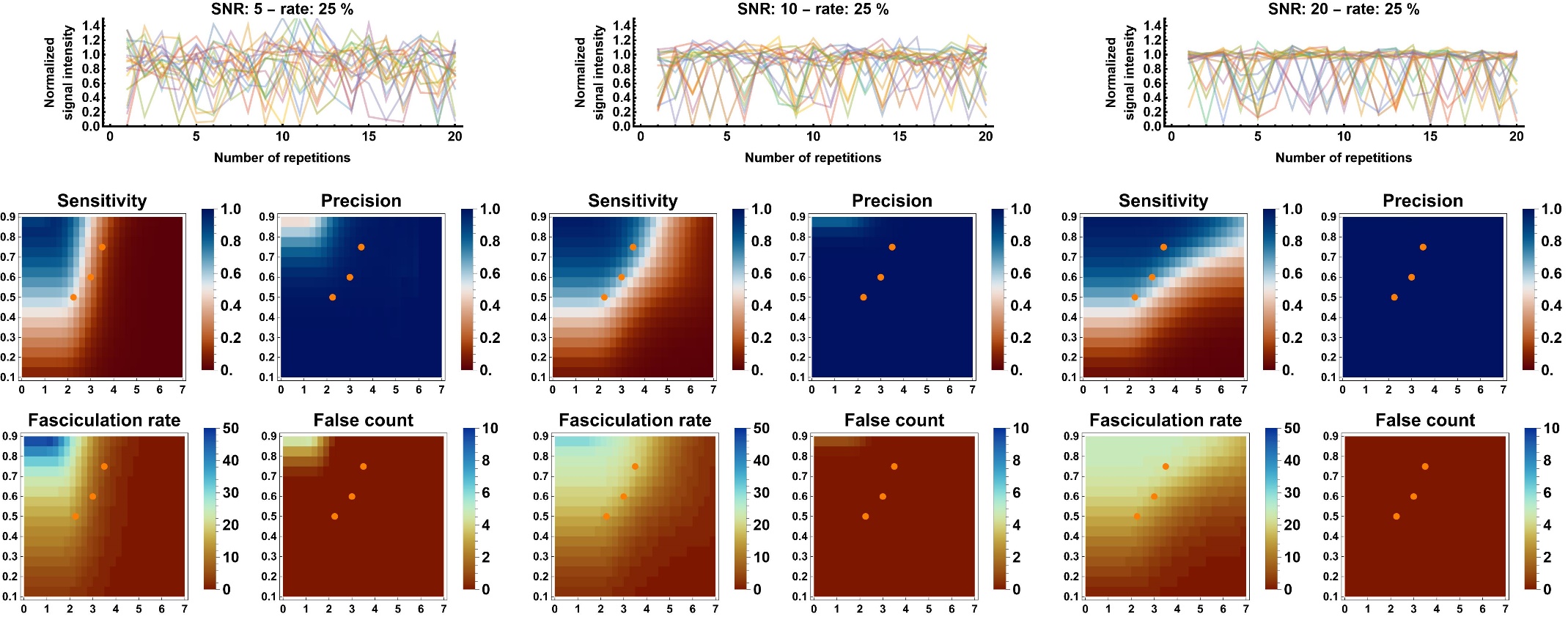


**Figure S8:** Monte-Carlo simulations for a single voxel in muscle tissue with an extremely high fasciculation rate (25%).

#### Muscle tissue with an extremely high fasciculation rate (50%), where algorithm fails.

Precision remains high, but sensitivity is 40% or lower for all SNR levels. This example shows that when the single voxel fasciculation rate becomes too high, the algorithm misses true fasciculations because it misclassifies fasciculations as pulsation.


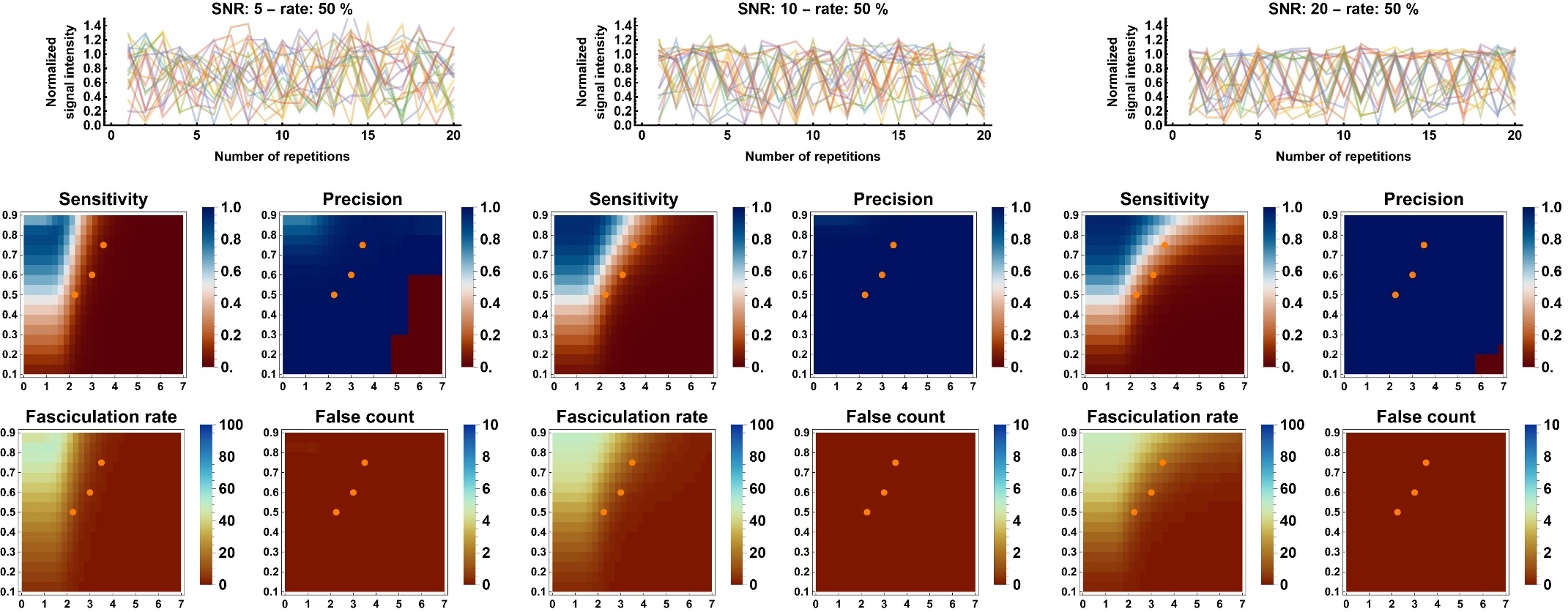


**Figure S9:** Monte-Carlo simulations for a single voxel in muscle tissue with an extremely high fasciculation rate (50%).

## Interrater comparison

The number of true positives, false positives and false negatives between rater 1 and rater 2 were compared with a Bland-Altman plot. The bias between rater 1 and rater 2 is 1 fasciculation or less for true positives, true negatives and false negatives. This was independent of the number of detected fasciculations. These findings indicate that rater 1 assigns more detected signal voids as true fasciculations compared to rater 2, and also assigns more undetected fasciculations as true fasciculations compared to rater 2. In other words, rater 1 was slightly less conservative in accepting signal voids as fasciculations compared to rater 2.


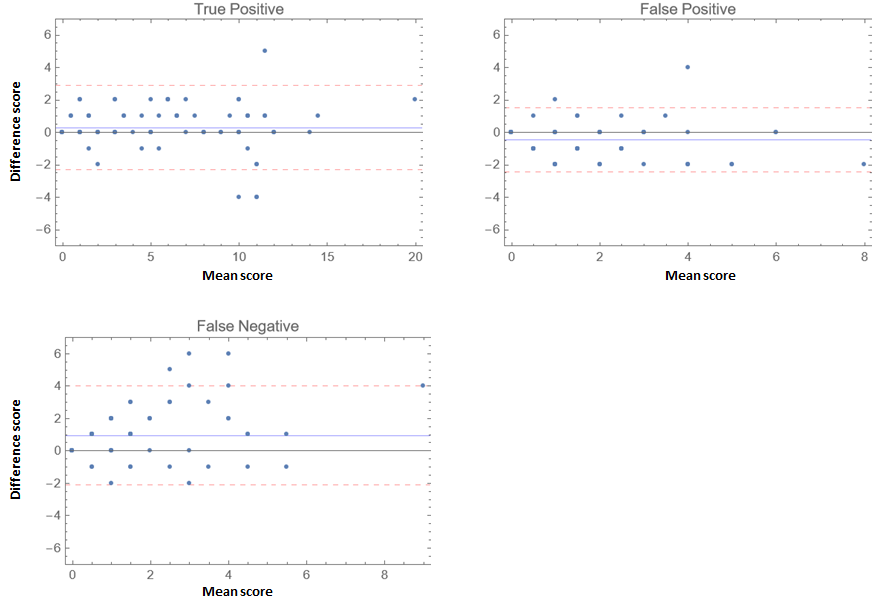


**Figure S10:** Bland-Altman plots comparing rater 1 and rater 2. Top left: True positives. Top right: False positives. Bottom left: False negatives.

## Effect of b-value on the fasciculation pattern in the healthy cohort


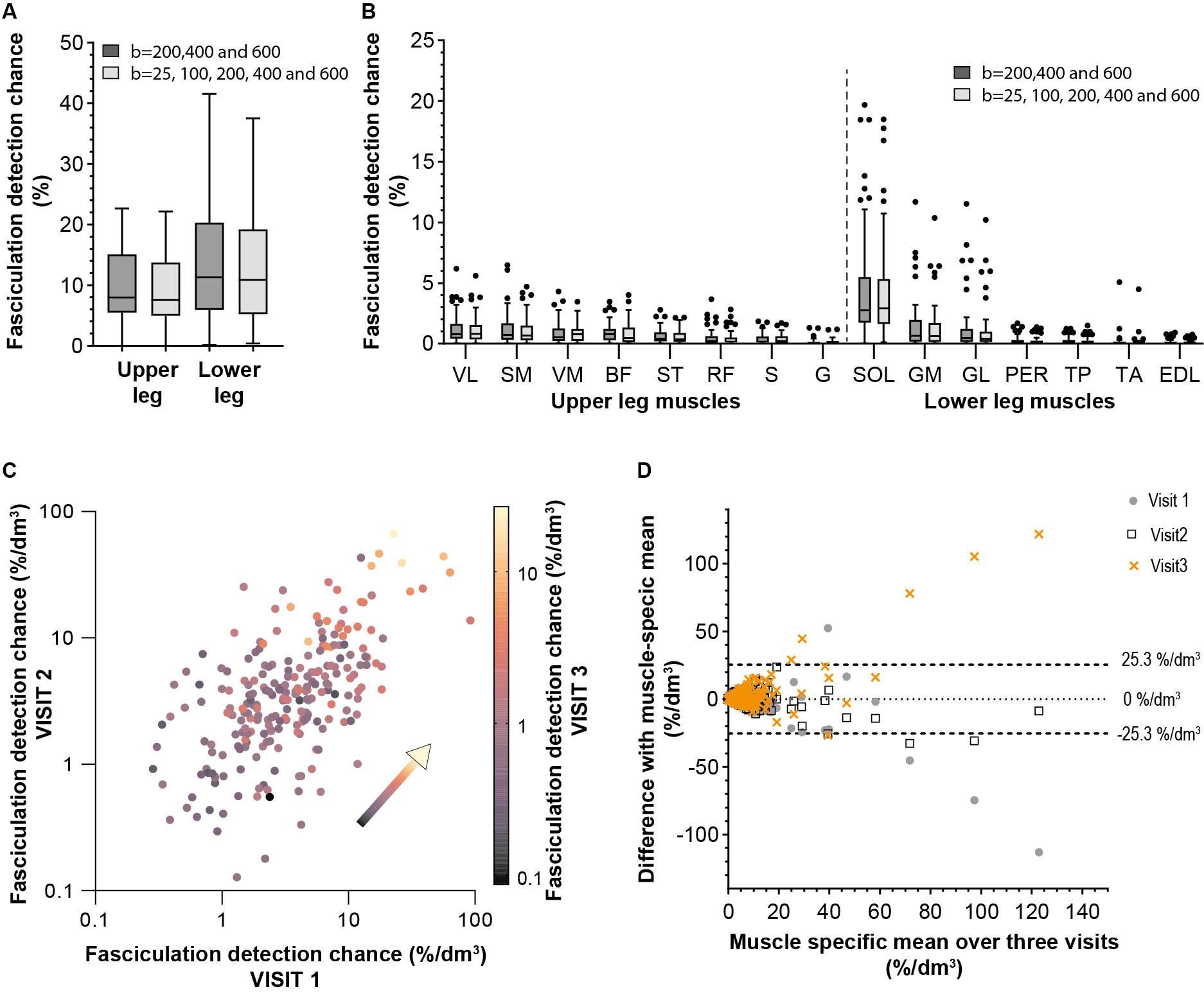


**Figure S11: The effect of including b-values 25, 100, 200, 400 and 600 s/mm^2^ instead of only 20, 400 and 600 s/mm^2^ on fasciculation pattern.** A-B) Fasciculation detection chance for b-value 200 s/mm^2^ and higher (dark grey) and b-value 25 s/mm^2^ and higher (light grey) for upper vs lower leg muscles (**A**) and for the individual muscles (**B**). For the upper leg *vs.* lower leg comparison, a whole-compartment mask was used including all muscles, and for the individual muscle analysis the individual muscle masks were used. C) Colour correlation plots of visit plot of visit 1 *vs.* visit 2 *vs.* visit 3 for DTI data including b-values of 25 s/mm^2^ and higher. The arrow displays the direction of the correlation. D) Jones plot for DTI data including b-values of 25 s/mm^2^ and higher depicting the muscle-specific mean over the three visits against the difference between the muscle-specific value at each visit and the muscle-specific mean over the three visits (visit 1: grey dots, visit 2: black open squares and visit 3: orange cross). The dotted line is the bias and the dashed lines are the limits of agreements.
