# Supplementary material for: Fasciculation distribution in a healthy population assessed with diffusion tensor imaging
Source: Physiol Rep. 2025 Mar 22;13(6):e70247. doi: 10.14814/phy2.70247 (PMC11928744; doi:10.14814/phy2.70247)
Supplement: Supplementary file 2 — Video S1. [file PHY2-13-e70247-s002.zip › Video S1A-D_caption.docx]

Video S1A: Video of the DTI acquisition pre-normalization showing a decrease in signal intensity in the muscle tissue with increasing b-value. Several signal voids can be observed.

Video S1B: Video of the DTI acquisition post-normalization showing a stable signal intensity in the muscle tissue with increasing b-value. Several signal voids can be observed.

Video S1C: Video of the MUMRI acquisition pre-normalization a stable signal intensity in the muscle tissue with increasing b-value. Several signal voids can be observed.

Video S1D: Video of the MUMRI acquisition post-normalization a stable signal intensity in the muscle tissue with increasing b-value. Several signal voids can be observed.
